# Conservative versus liberal oxygen therapy for intensive care unit patients: meta-analysis of randomized controlled trials

**DOI:** 10.1186/s13613-024-01300-7

**Published:** 2024-04-26

**Authors:** Xin-yu Li, Bing Dai, Hai-jia Hou, Hong-wen Zhao, Wei Wang, Jian Kang, Wei Tan

**Affiliations:** Department of Respiratory and Critical Care Medicine, The First Affiliated Hospital of China Medical University, No.155, Nanjing North Street, Heping District, Shenyang, China

**Keywords:** Conservative oxygen therapy, Liberal oxygen therapy, Mortality, ICU

## Abstract

**Background:**

It remains unclear whether conservative oxygen therapy (COT) or liberal oxygen therapy (LOT) is more beneficial to the clinical outcomes of intensive care unit (ICU) patients. We systematically reviewed the efficacy and safety of conservative versus liberal oxygen therapy for ICU patients.

**Methods:**

We systematically searched PubMed, Embase, Web of Science, Scopus, Cochrane Central Register of Controlled Trials, ClinicalTrials.gov, MedRxiv, and BioRxiv for reports on randomized controlled trials (RCTs) that compared the effects of COT versus LOT on the clinical outcomes of ICU patients published in English before April 2024. The primary outcome was the mortality rate, secondary outcomes included ICU and hospital length of stay, days free from mechanical ventilation support (MVF), vasopressor-free time (VFT), and adverse events.

**Results:**

In all, 13 RCTs involving 10,632 patients were included in analyses. Meta-analysis showed COT did not reduce mortality at 30-day (risk ratio [RR] = 1.01, 95% confidence interval [CI] 0.94 to 1.09, I^2^ = 42%, P = 0.78), 90-day (RR = 1.01, 95% CI 0.95 to 1.08, I^2^ = 9%, P = 0.69), or longest follow-up (RR = 1.00, 95% CI 0.95 to 1.06, I^2^ = 22%, P = 0.95) compared to LOT in ICU patients. In subgroup analyses, no significant difference was observed between the two groups in terms of the different ICU, baseline P/F, and actual PaO_2_. In addition, COT did not affect ICU length of stay, hospital length of stay, or VFT, it only affected MVF days.

**Conclusions:**

COT did not reduce all-cause mortality in ICU patients. Further RCTs are urgently needed to confirm the impact of COT strategy on specific populations.

**Supplementary Information:**

The online version contains supplementary material available at 10.1186/s13613-024-01300-7.

## Introduction

Oxygen is essential for human survival and plays a crucial role in a wide range of physiological processes [1]. Oxygen therapy is among the most common interventions in critical illnesses [2]. However, excessive oxygenation can enhance the production of reactive oxygen species, which can lead to oxidative damage to cellular components, including DNA, lipids, and proteins, ultimately resulting in cell death, producing inflammation that damages lung tissue further [3–5]. Although previous studies in volunteers and experimental models have investigated the detrimental effects of hyperoxia [6, 7], overuse of oxygen remains prevalent in intensive care unit (ICU) particularly among patients with hypoxemic respiratory failure [8–10].

Therefore, conservative oxygen therapy (COT) has been proposed in recent years to prevent excessive oxygen exposure to patients. In 2016, a single-center, open-label randomized controlled trial (RCT) indicated that a conservative protocol resulted in lower ICU mortality [11]. Two years later, the IOTA systematic review and meta-analysis suggested that a LOT strategy above a peripheral oxygen saturation (SpO_2_) range of 94–96% is associated with increased mortality; this result supported the conservative administration of oxygen therapy in acutely ill patients [12]. However, such findings were not supported by subsequent studies, which yielded conflicting results with a similar setup [13–24]. Despite recommendations for oxygenation targets [25, 26], the clinical efficacy of oxygenation strategies in critically ill patients remains uncertain.

As new trial data have been published recently [27, 28], we present an updated this review of this topic. In addition, due to the heterogeneity of patient characteristics in relation to different types of ICU, baseline oxygenation, and actual target oxygenation, it is difficult to interpret the results of these systematic reviews using pairwise meta-analysis [12–24]. It is essential to evaluate the target oxygenation and distinguish subpopulations who are likely to benefit from different oxygenation strategies.Accordingly, we focus on data from mixed ICUs vs. medical ICUs, different baseline P/Fratios (P/F), and actual target arterial partial pressure of oxygen (PaO_2_), and conduct a systematic review and meta-analysis of the RCTs. Specifically, we compared COT versus LOT in those subpopulations, to explore the optimal oxygenation targets in ICU patients.

## Materials and methods

We designed and and wrote this report the study according to the Preferred Reporting Items for Systematic Reviews and Meta Analysis Protocols checklist (PRISMA-P) [29] (the checklist is presented in Additional file 1: S6) and the principles of the Cochrane Handbook including the Methodological Expectations of Cochrane Intervention Reviews standards [30, 31]. We used GRADEpro GDT to assess the certainty of the results [32, 33]. Trial sequential analysis (TSA) [34, 35] was performed by using TSA version 0.9.5.10 to further investigate the effects of COT and LOT, which was achieved by defining the required information size, using the O’Brien-Fleming boundaries to adjust the thresholds for statistical significance each time a trial was included, and introducing the threshold for futility. Our protocol of this systematic review was pre-registered in the International Prospective Register of Systematic Reviews (PROSPERO: CRD42023434202).

### Search strategy

Two authors (XYL and WT) independently searched PubMed, Embase, Web of Science, Scopus, Cochrane Central Register of Controlled Trials, ClinicalTrials.gov, MedRxiv, and BioRxiv before April 2024, focusing on adult ICU patients subjected to two different oxygenation strategies. The details of the search strategy are summarized in additional files to this report (Additional file 1: Search strategy). We searched RCTs in English for which full texts were available. All of the references listed in the included studies were reviewed, and the relevant studies were manually searched.

### Inclusion and exclusion criteria

Eligible studies were those that met the following criteria: (1) the enrolled adult patients were admitted to an ICU; (2) patients were randomly assigned to a COT group or a LOT group; (3) oxygenation targets between the two groups were realized by PaO_2_, arterial oxygen saturation (SaO_2_), or SpO_2_ rather than constant fraction of inspired oxygen (FiO_2_), we did not determine a *priori* thresholds of oxygenation for the two groups to ensure inclusion of all relevant trials; and (4) mortality was included as a primary or secondary outcome. Exclusion criteria were as follows: (1) the patients did not meet screening criteria; (2) only included patients at risk for ischemia or hypoxic encephalopathy (including traumatic brain injury, stroke, myocardial infarction, and cardiac arrest) or who underwent surgery (including trauma and coronary artery bypass surgery) were included; and (3) the publication was not in English; was a conference reports, commentary, and reviews; and/or represented a redundant publication from a single study.

### Outcomes and definition

The primary outcome of interest was 30 (including 28)-day mortality, 90-day mortality, and the longest follow-up mortality in each study.We performed sensitivity analyses according to the two different ICUs (mixed/medical). A mixed ICU was defined as a unit that included both medical and surgical patients, whereas a medical ICU was one that included medical patients. We also performed a subgroup analysis from different baseline P/F at enrollment (mild to moderate hypoxemia, P/F ≥ 150 mmHg; moderate to severe hypoxemia, P/F < 150 mmHg), and the actual PaO_2_ ( PaO_2_ in the COT group ≥ 80 mmHg, < 80 mmHg). The secondary outcomes were ICU length of stay, hospital length of stay, days free from mechanical ventilation support (MVF), vasopressor-free time (VFT), and adverse events.

### Data extraction and quality assessment

Two investigators (XYL and WT) independently extracted and recorded the desired information from the included studies based on the Cochrane recommendations [28], consisting of the first author, year of publication, setting, country, sample size, intervention protocols, demographic and illness characteristics of patients, and study outcomes. Authors were contacted in cases of missing data or if the reporting format was not suitable for the meta-analysis (e.g., included the data of surgical patients and medical patients). Datas were extracted using the software (GetDataW) when presented in a figure in the trial, or in part from secondary analysis of other studies. Continuous datas were extracted as sample size and mean (standard deviation, SD) or median (inter quartile range, IQR) provided in the studies, with the conversion of medians to estimated mean (SD). Any discrepancies that arose were resolved by the involvement of a third author (BD or HJH).

To evaluate the quality of the eligible RCTs, we used the risk of bias tool recommended by the Cochrane Collaboration [29]. The potential sources of bias were rated according to the following items: sequence generation, allocation concealment, blinding, incomplete outcome data, selective outcome reporting, and other sources of bias. Besides, the quality of evidence was assessed according to the GRADE (Grading of Recommendations Assessment, Development and Evaluation) guidelines on the basis of study limitations, imprecision, inconsistency, indirectness and publication bias for the targeted outcomes [33]. Publication bias was evaluated by visually inspecting funnel plots.

### Statistical analysis

Meta-analysis and forest plots were performed using the Cochrane systematic review software Review Manager (RevMan; version 5.4.1; The Nordic Cochrane Center, The Cochrane Collaboration, Copenhagen, Denmark). Dichotomous outcomes are expressed as RRs with 95% CIs, while continuous outcomes are expressed as weighted MDs and 95% CIs, mean (SD) were estimated from median IQRs for further comparison. Heterogeneity was tested using I^2^ statistics. A fixed-effect model was applied when I^2^ < 50%, indicating insignificant heterogeneity, whereas a random-effects model was chosen for cases of significant heterogeneity (I^2^ > 50%). P < 0.05 was considered statistically significant. A relative risk reduction (RRR) of 20%, a type I error level of 5%, and a type II error level of 10% were used in TSA.

## Results

### Study selection

**Fig. 1:**
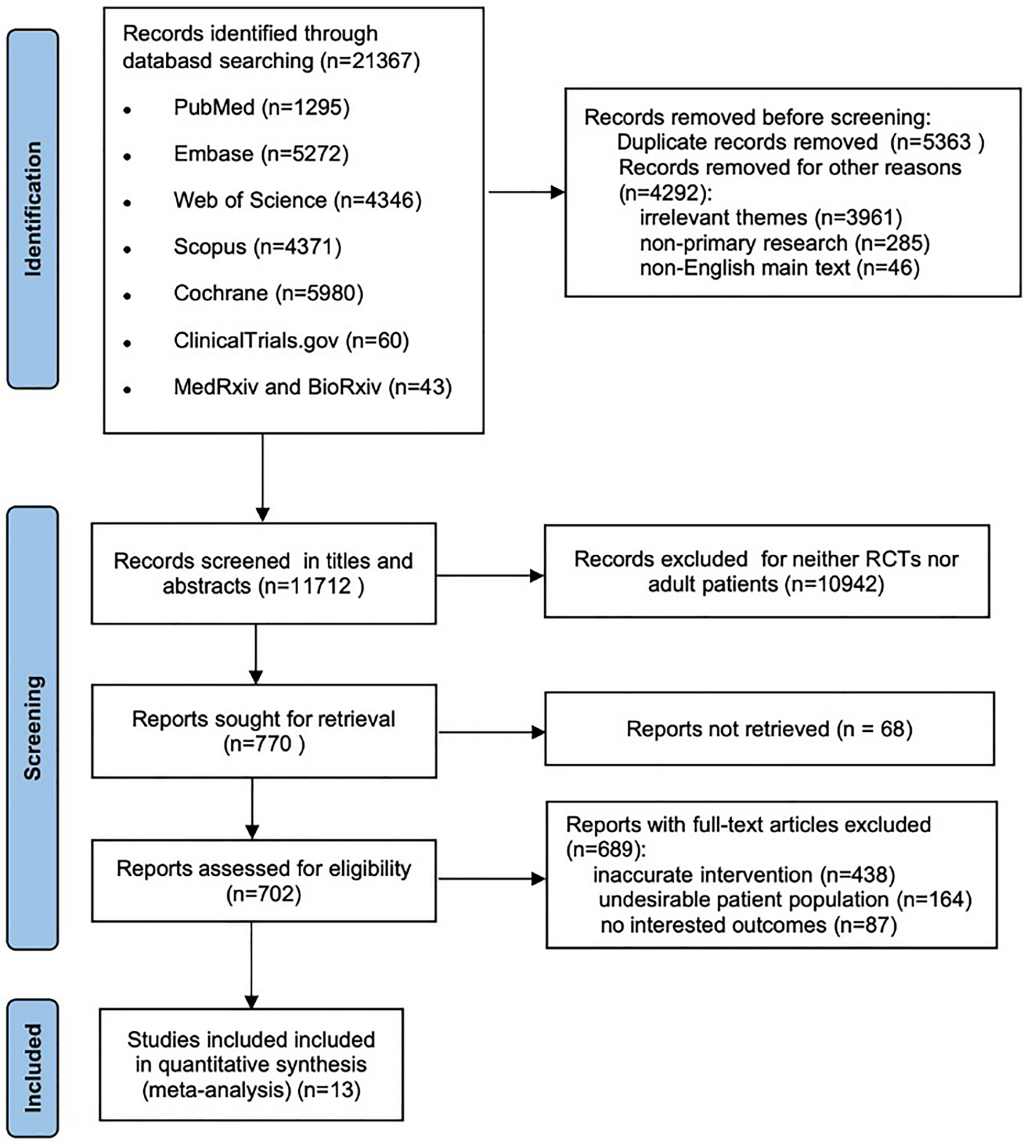
Study flow diagram

Following the search strategy, 21,367 records were imported for screening, and 11,712 records were screened by titles and abstracts after removal of duplicates or other reasons. Of these, 10,942 studies were excluded for either not being RCTs or not including adult patients. Furthermore, 68 reports could not be retrieved for evaluation. The remaining 702 studies underwent full-text assessment, of which 689 were excluded for the following reasons: inaccurate intervention, undesirable patient population, or presenting no outcomes of interest. Thus, 13 eligible RCTs [11, 27, 28, 36–45] were ultimately involved in the meta-analysis as depicted in Fig. 1. Two post-hoc subgroup analyses [46, 47] of the HOT-ICU trial [39] were also included for subgroup analyses.

### Study description and quality assessment

The main characteristics of the 13 RCTs and 2 post-hoc subgroup analyses are summarized in Table 1, and further demographic details are shown in Additional file 1: Tables S1 and S2. All RCTs were performed in ICU; 9 were conducted in mixed ICU settings [11, 27, 36–39, 41–43] and 6 were conducted in medical ICU settings [28, 40, 44–47]. The mean baseline P/F varied among the included studies, being ≥ 150 mmHg in 8 studies [11, 27, 36, 38, 40, 42–44] and < 150 mmHg in 6 studies [28, 39, 41, 45–47]. The mean actual PaO_2_ levels in both COT and LOT group also varied among the included studies. In the COT group, it ranged from 61 to 87 mmHg, being ≥ 80 mmHg in 5 studies [11, 37, 38, 42, 43], and < 80 mmHg in 10 studies [27, 28, 36, 39–41, 44–47]. In the LOT group, it ranged from 76 to 115 mmHg in 15 studies [11, 27, 28, 36–47]. Regarding the difference in mortality, the rate was 5% lower in the COT group than in the LOT group in 3 studies [11, 37, 42], within 5% between the two groups in 8 studies [27, 28, 36, 38, 39, 43, 44, 46] and 5% higher in the COT group than in the LOT group in 4 studies [40, 41, 45, 47].

**Table 1:** Characteristics of the studies included

Study	Country	Study design	Group	Simple size (Total, Medical, Surgical, n)	Baseline PaO_2_/FiO_2_ (mmHg)	Targeted SpO_2_,% (PaO_2_, mmHg)	Actual PaO_2_^#^ (mmHg)	Mortality (30-,90-day,%)
Mortality (LOT-COT > 5%)								
Asfar 2017 [42]	France	Multicenter RCT	COT	223, ND	228 ± 103	88–95	84.3 ± 20.2	35.5%, 41.5%
			LOT	219, ND	220 ± 103	FiO_2_:1.0	87.3 ± 22.4	42.9%, 47.9%
Girardis 2016 [11]	Italy	Single-center, open-label RCT	COT	236, 77, 139	≥ 150	94–98 (70–100)	87.7 ± 13.4	16.2%, ND
			LOT	244, 86, 132	≥ 150	97–100 (up to 150)	102.0 ± 20.9	25.2%, ND
Yang 2019 [37]	China	Multicenter RCT	COT	100, 88, 12	≥ 100	90–95	84.7 ± 21.1	26.0%, ND
			LOT	114, 94, 14	≥ 100	96–100	97.7 ± 27.8	32.5%, ND
Mortality (LOT-COT − 5% ~ 5%)								
Gelissen 2021 [43]	Netherlands	Multicenter RCT	COT	294, 143, 50	PaO_2_:92.8 ± 36.4, FiO_2_:0.48 ± 0.16	ND (60–90)	81.4 ± 12.5	ND, 35.1%
			LOT	280, 139, 41	PaO_2_:93.8 ± 24.6, FiO_2_:0.49 ± 0.15	ND (105–135)	96.5 ± 22.4	ND, 34.4%
Mackle 2020 [38]	Australia and New Zealand	Multicenter RCT	COT	499, 335, 149	259 ± 146	91–96	83.1 ± 2.8	31.8%, 34.7%
			LOT	501, 335, 146	245 ± 138	≥ 91	94.9 ± 4.5	29.1%, 32.5%
Nielsen 2024 [28]	Denmark, Switzerland, Norway, Iceland, and Wales	Multicenter RCT	COT	365, 362, 0	94.7 ± 38.0	ND (60)	71.3 ± 5.2	30.2%
			LOT	361, 358, 0	98.0 ± 37.2	ND (90)	90.3 ± 8.9	34.7%
Panwar 2016 [36]	Australia, France and New Zealand	Multicenter, multinational RCT	COT	53, 39, 10	248 ± 112	88–92	70.3 ± 3.8	ND, 40.4%
			LOT	51, 41, 8	247 ± 113	≥ 96	92.3 ± 5.3	ND, 37.3%
Rasmussen [46]^*^	Substudy of HOT-ICU [39]		COT	54, 54, 0	109.3 ± 49.7	ND (60)	70.8 ± 4.6	ND, 40.7%
			LOT	56, 56, 0	100.5 ± 41.7	ND (90)	92.0 ± 8.0	ND, 41.8%
Schjørring 2021 [39]	Denmark, Switzerland, Finland, Netherlands, Norway, UK and Iceland	Multicenter RCT	COT	1462, 1248, 205	121.6 ± 51	ND (60)	71.3 ± 7.3	ND, 42.9%
			LOT	1466, 1240, 217	120.4 ± 47.3	ND (90)	93.0 ± 8.6	ND, 42.4%
Semler 2022 [44]	USA	Cluster-crossover RCT	COT	963^a^, ND 992^b^, ND	PaO_2_:97.7 ± 58.7, FiO_2_:0.32 ± 0.18^a^ PaO_2_:107.7 ± 64.6,FiO_2_:0.37 ± 0.19^b^	88–92 (55–65)^a^, 92–96 (65–80)^b^	70.8^a^ 82.7^b^	34.8%, ND 34.0%, ND
			LOT	1032^c^, ND	PaO_2_:103.7 ± 62.4,FiO_2_:0.48 ± 0.15^c^	96–100 (> 80)^c^	90.2^c^	33.2%, ND
van der Wal 2023 [27]	Netherlands and Italy	Multicenter RCT	COT	439, 258, 76	≥ 150	91–94 (55–80)	76.1 ± 10.2	38.5%, 43.0%
			LOT	443, 251, 78	≥ 150	96–100 (110–150)	114.8 ± 21.4	34.7%, 40.4%
Mortality (COT-LOT > 5%)								
Barrot 2020 [41]	France	Multicenter RCT	COT	103, ND	116.8 ± 47.4	88–92 (55–70)	71.3 ± 3.2	34.3%, 44.4%
			LOT	102, ND	120.1 ± 53.6	≥ 96 (90–105)	100.6 ± 6.7	26.5%,30.4%
Klitgaard [47]^*^	Substudy of HOT-ICU [39]		COT	82, 82, 4	120.8 ± 53.2	ND (60)	72.5 ± 10.2	ND, 65.4%
			LOT	86, 86, 6	119.0 ± 53.2	ND (90)	95.3 ± 7.4	ND, 54.6%
Martin 2021 [40]	England	Single-center RCT	COT	17, 17, 0	PaO_2_:86.3 ± 18.0 FiO_2_:0.43 ± 0.12	88–92	65.0 ± 8.5	31.3%, 40.0%
			LOT	17, 17, 0		No restrictions	90.3 ± 22.4	29.4%, 31.3%
Nafae 2023 [45]	Egypt	Single-center RCT	COT	28, 28, 0	129.36 ± 32.6	88–92	61.4 ± 3.1	ND, 35.7%
			LOT	28, 28, 0	120.36 ± 28.63	94–97	76.7 ± 5.4	ND, 21.4%

UK: United Kingdom; USA: United States of America; RCT: randomized controlled trial; COT: conservative oxygen therapy; LOT: liberal oxygen therapy; ND: No date

^a^In a lower SpO_2_ target (90%; goal range, 88 to 92%)

^b^In an intermediate SpO_2_ target (94%; goal range, 92 to 96%)

^c^In a higher SpO_2_ target (98%; goal range, 96 to 100%)

^*^Substudy of HOT-ICU [39]

^#^Actual PaO_2_ obtained for the longest follow-up (72 h-90 days) included in the study

The results of quality assessment of the included studies are shown in Figure S1 (Additional file 1: Fig. S1). No selection bias was found in 13 studies, but high performance bias was found due to their unblinded designs. Symmetrical funnel plots of mortality rate showed no significant publication bias (Additional file 1: Fig. S2).

### Outcomes

**Fig. 2:**
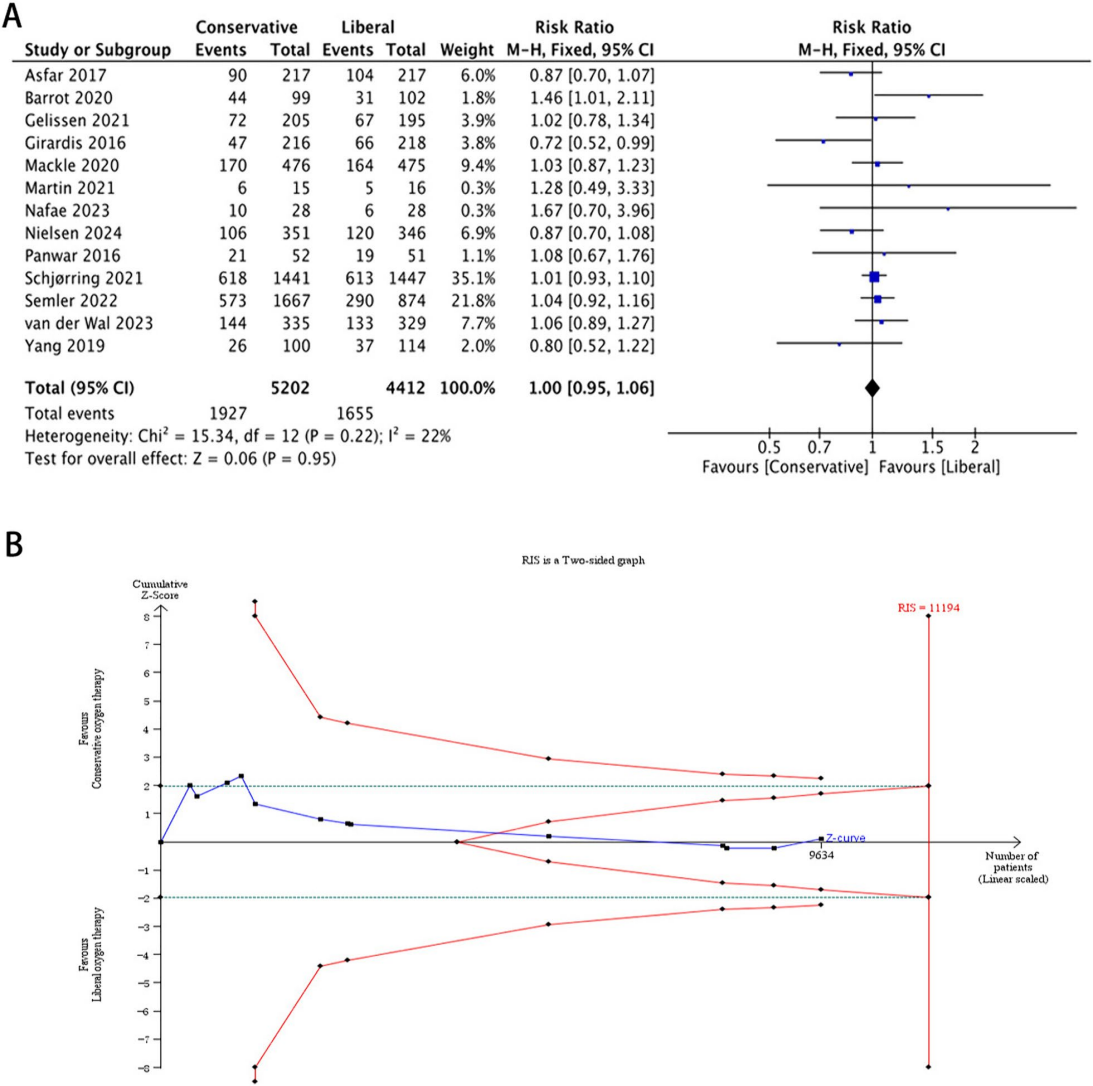
Mortality at the longest follow-up and TSA of the included studies. **A** Mortality at the longest follow-up and **B** TSA of the the included studies

All 13 RCTs [11, 27, 28, 36–45] (10,632 patients) reported mortality outcomes: 4 reported 30-day mortality [11, 37, 44, 45]; 5 reported both 30- and 90-day [27, 38, 40–42] (and 180-day [38]) mortality; and 4 reported 90-day mortality [28, 36, 39, 43]. The mortality at the longest follow-up in the COT and LOT groups was 37.04% (1927 of 5202 patients) and 37.51% (1655 of 4412 patients), respectively, with no significant differences between the two groups (Fig. 2A). The results of the TSA was shown in Fig. 2B. The cumulative Z curve crossed the futility boundary but not crossed the conventional boundary, and 85.89% (9,614 of 11,194 patients) of the required information size was accrued. The results indicated that, when compared with LOT, COT did not reduce the relative risk of the longest follow-up mortality by 20% among ICU patients. The certainty of the evidence was very low (GRADE, Additional file 1: Fig. S3). Also, no significant differences were observed in the analysis of 30-day mortality [11, 27, 37, 38, 40–42, 44, 45] or 90-day mortality [27, 28, 36, 38–43] between the two groups (Additional file 1: Fig. S4, S5).

**Fig. 3:**
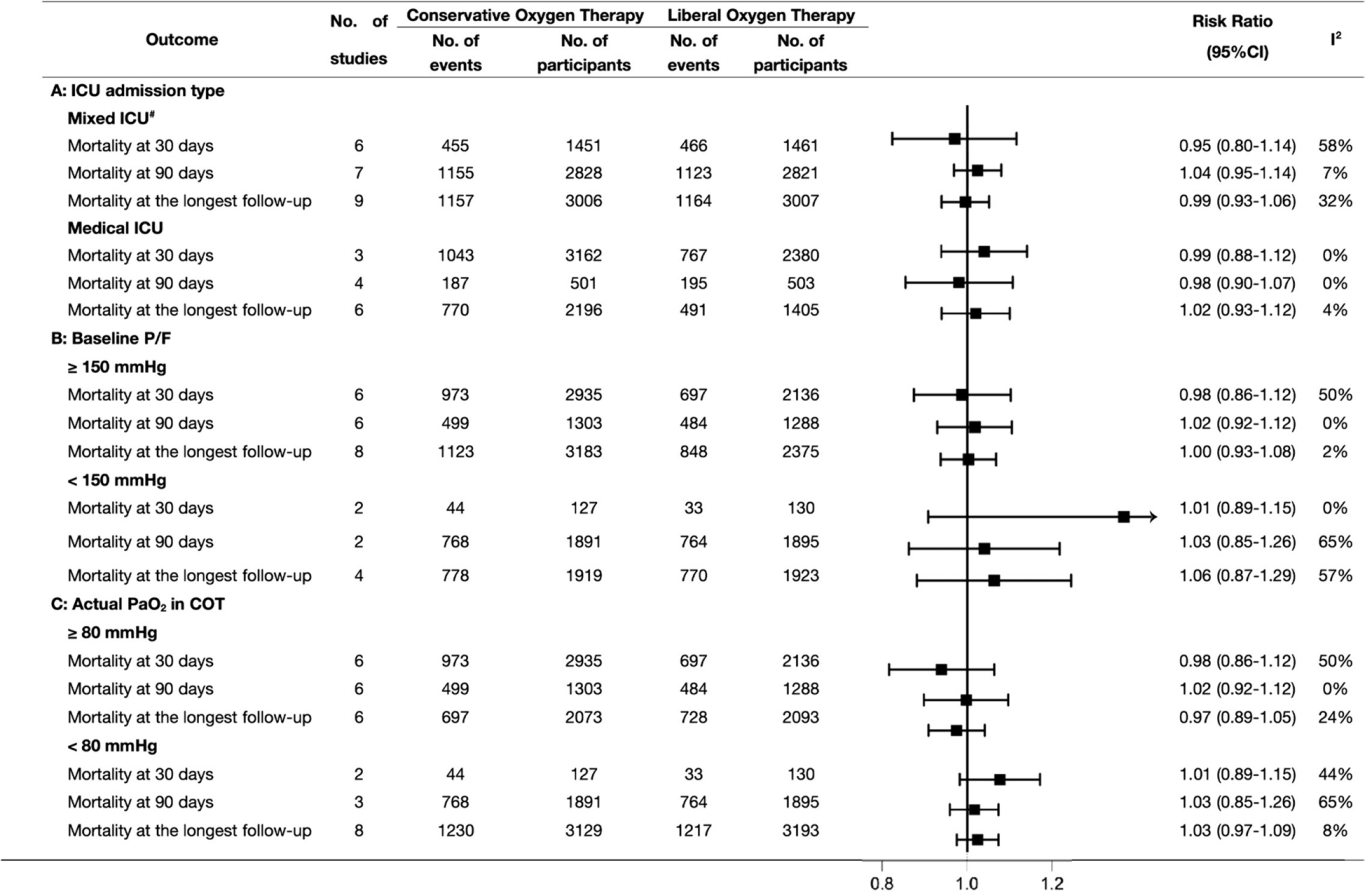
Subgroup analyses of mortality

In subgroup analyses, there were no significant differences in mortality at any analyzed time point (30-day, 90-day, longest follow-up) between the two groups in terms of ICU admission type, different baseline P/F, or different actual PaO_2_ (Fig. 3; Additional file 1: Fig. S6, 7). Further details of the TSA results were shown in Additional file 1: Fig. S8–10.

No differences were found in terms of the ICU length of stay [11, 27, 36, 40, 42, 43, 45], length of hospital stay, [11, 27, 36, 40], or VFT days [36, 38, 42, 44] between the COT and LOT group, but MVF days [11, 27, 28, 36, 38, 42–44] was significant longer in the LOT group than in the COT group (Additional file 1: Fig. S11–13). The certainty of the evidence was low to very low (Additional file 1: Fig. S3).

**Fig. 4:**
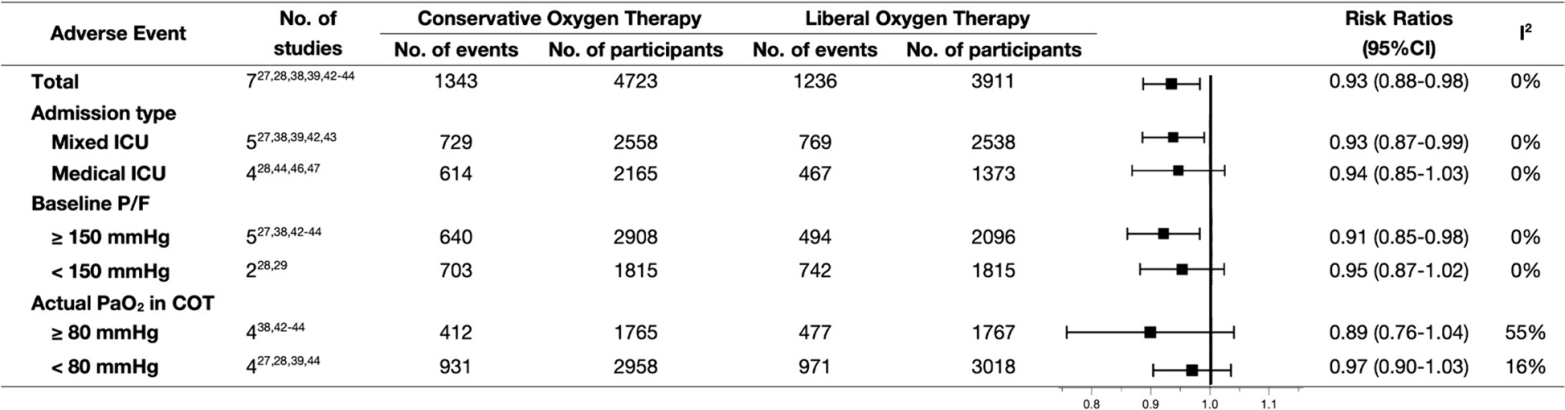
Overall and subgroup analyses of adverse events

Adverse events were reported in 7 RCTs [27, 28, 38, 39, 42–44], including organ failure, shock, infection, ICU-acquired weakness, seizure, and delirium (Additional file 1: Table S3). The incidence of adverse events was significantly lower in the COT group than in the LOT group. The results of TSA indicated that the improvement of COT could be considered conclusive with the available evidence (Additional file 1: Fig. S14). However, the certainty of the evidence was low (Additional file 1: Fig. S3). In the subgroup analyses, the incidence of adverse events in the COT group was significantly lower among the patients who enrolled in mixed ICU settings and with baseline P/F ≥ 150 mmHg. However, no differences were found between the two oxygenation strategies for patients enrolled in medical ICU settings and with baseline P/F < 150 mmHg (Fig. 4, Additional file 1: Fig. S13).

## Discussion

In this systemic review and meta-analysis, we found that COT did not reduce the mortality rate relative to the LOT group in ICU patients; this was also true in terms of the different ICUs, baseline P/F, and actual PaO_2_ in the subgroup analyses. Some studies showed a slight trend of higher mortality in the COT group with actual PaO_2_ < 80 mmHg and in medical ICU settings. COT did not affect ICU length of stay, hospital length of stay, or VFT, only MVF days. The incidence of adverse events was significantly lower in the COT group (among patients enrolled in mixed ICU settings and with baseline P/F ratio ≥ 150 mmHg) than in the LOT group, but no differences were found between the two oxygenation strategies for patients enrolled in medical ICU settings and with baseline P/F < 150 mmHg.

Our review has several strengths. First, we included studies only involving ICU patients, and new high-quality RCTs were included [27, 28]. Second, we performed TSA with adjusted CIs in order to control for risk of random errors due to multiple outcomes, sparse data, and repetitive testing on accumulated data, to evaluate the benefits and harms of COT versus LOT in critically ill patients. We also contacted relevant trial authors if additional information was required. Third, to explore the robustness of our results and the influence of hypoxemia severity, ICU population and actual oxygenation on our primary outcome, we have performed the subgroup analyses according to ICU population, baseline oxygenation, and actual PaO_2_.

In this review, COT did not reduce mortality of ICU patients. In recent meta-analyses summarized in Additional file 1: Table S5 [12–24, 48], only two earlier reviews found that COT may result in a lower mortality rate. An IOTA meta-analysis [12] was conducted in both ICUs and ordinary care settings, considering all types of diseases, and it suggested that a LOT strategy above an SpO_2_ range of 94–96% was associated with increased mortality, but subgroup analyses revealed no significant differences in critical care patients. Another meta-analysis that included ICU patients in only four RCTs found that COT resulted in significantly lower mortality rate [48]. However, such findings have not been supported by any subsequent meta-analyses published after 2019 [13, 24]. Our systematic review including more RCTs further confirms these results. However, because blinding of participants and/or personnel is not possible, the the certainty of the evidence was low. Still, the TSA indicated that over 80% of the required information size was accrued, and evidence was able to assess the benefit or harm between the two groups.

No differences of mortality were found between two oxygenation strategies for patients enrolled in medical and mixed ICU settings. Compared to the previous meta-analyses [13, 24], we included more studies in the subgroup analysis. However, the TSA indicated that in most subgroups, the samples did not reach the required information size. We noticed that there was a difference in mortality of up to 5% between the two groups in some studies (Table 1). Interestingly, studies showing a trend of higher mortality in the COT group were mostly conducted in medical ICU settings [40, 41, 45, 47], and the main diagnoses of the included patients were medical diseases, mainly respiratory diseases (Additional file 1: Table S1). On the contrary, studies showing a trend of lower mortality in the COT group were all in mixed ICU settings [11, 37, 42]. We summarized the comorbidities of patients located in medical and mixed ICU and found that the incidence of cardiovascular, respiratory, and digestive diseases was significantly higher in medical ICU patients than that in mixed ICUs (Additional file 1: Table S4). It is reasonable to assume that the patients in medical ICUs may have had more severe gas-exchange impairments and refractory hypoxemia, requiring more oxygen. It may also be worth noting that the COT strategy avoids hyperoxemia but exposes patients to a higher risk of hypoxia, especially in these patients with more comorbidities [26]. Clinical trials comparing different oxygenation groups for these specific patient groups are needed; if possible, such studies should also incorporate stratification of important baseline risk factors (e.g., comorbidities).

In most including RCTs, SpO_2_ has been the primary parameter defining the target oxygenation range, but discrepancies sometimes exist between the targeted goals and the actual levels. Actually, PaO_2_ is superior to defining oxygenation target levels precisely and minimizing overlap between two groups [49]. Zhao et al. [17] performed a systematic review according to oxygenation goals, and found that different oxygenation goals do not lead to different mortalities in mechanically ventilated critical ill patients. Further, we performed the subgroup analysis based on the actual PaO_2,_ and no differences of mortality were found between COT and LOT. However, we noticed a trend that (Table 1), the COT group with lower actual PaO_2_ (< 80 mmHg) may have a higher mortality rate than that of LOT group in some studies [40, 41, 45, 47]; while the COT group with higher PaO_2_ (≥ 80 mmHg) may have lower mortality in some studies [11, 37, 42], and the actual PaO_2_ was basically equal to the LOT group in some other studies (Table 1). As the normal range for PaO_2_ at sea level in healthy individuals is 80 to 100 mmHg [50], the COT strategy may not represent permissive hypoxia, which has not been well studied in adults. The observed degree of difference in mortality may have clinical significance, and thus more careful oxygen titration with “permissive hypoxia” should be considered in these patients until more robust evidence is available.

COT was not associated with any advantages in terms of ICU length of stay, hospital length of stay, or VFT compared to LOT, only MVF days. We believe that many factors contributed to these results, such as the primary diseases of patients admitted to different ICUs, the severity of baseline disease, and the treatment effects.

We also found that the incidence of adverse events in LOT was significantly higher among patients enrolled in mixed ICU settings and with a baseline P/F ≥ 150 mmHg than in COT. This is consistent with previous conclusions that higher oxygenation targets are associated with more adverse events [10]. However, no differences were found between the two oxygenation strategies for patients enrolled in medical ICU settings and with baseline P/F < 150 mmHg. This may also be due to the fact that these patients have more severe exchange impairments and refractory hypoxemia, where the adverse events are offset by the benefits of corrected hypoxia from oxygen therapy. However, due to the different definitions and the inadequate blinding of adverse events, more robust data are needed for a more compelling conclusion. We should also pay close attention to the microcirculation, long-term neurological function and others complications; new technological approaches, such as biomarkers, can be also considered in the future research.

There may be some implications for practice and future research. Given the presence of confounding factors in many existing RCTs, it remains of paramount importance to continue to conduct clinical trials, ideally comparing groups with a clinically relevant contrast between specific patient groups, such as according to the type and severity of disease of patients in the respiratory ICU; machine learning methods using data from these trials could also be utilized to build models for individual patients [51]. Meanwhile, the oxygenation strategies in all trials were grouped by PaO_2_, SpO_2_, or FiO_2_, all of which required manual adjustment during titration. New techniques, such as automated oxygen titration may better identify the suitable oxygenation target for a specific population in the future research [52]. Finally, adverse events are important signals for clinical practice guidelines; it may be necessary to take adverse events as primary or secondary outcomes, more robust data is needed for a compelling conclusion.

Some limitations of our review should be mentioned. First, the definitions of COT and LOT were not quite concordant among the studies assessed and the actual oxygenetions were also very different. Second, clinical heterogeneity among studies is a common concern. Third, inadequate blinding is often associated with performance bias. Finally, TSA indicated that the information size was insufficient for most outcomes, especially in most subgroups.

## Conclusion

In conclusion, this systematic review and meta-analysis found that COT did not reduce all-cause mortality at 30-day, 90-day or longest follow-up of ICU patients. There was a trend, but without a statistical difference, showing that patients in the COT group with lower PaO_2_ had an increased mortality rate in medical ICU settings; further studies are needed to confirm our findings. COT was associated with a lower incidence of adverse events among patients enrolled in mixed ICU settings and with baseline P/F ≥ 150 mmHg; however, no differences were found between the two oxygenation strategies for patients enrolled in medical ICU settings and with baseline P/F < 150 mmHg.

### Supplementary Information

**Additional file 1:  Search strategy.** **Table S1.** Demographic details of the included studies. **Table S2.** Primary diagnosis or comorbidities of the included studies. **Table S3.** Comorbidities of the included studies in medical ICU and Mixed ICU. **Table S4.** Adverse events of the included studies. **Figure S1.** Assessment on the risk of bias for the included RCTs. **Figure S2.** Funnel plot of mortality at the longest follow-up for the included studies. **Figure S3.** The GRADE assessment of the certainty of the evidence. **Figure S4.** Mortality at 30 days and TSA of the the included studies. **Figure S5.** Mortality at 90 days and TSA of the the included studies. **Figure S6.** Subgroup analysis of 30-day mortality for the included studies. **Figure S7.** Subgroup analysis of 90-day mortality for the included studies. **Figure S8.** TSA of 30-day mortality (subgroup analysis) for the included studies. **Figure S9.** TSA of 90-day mortality (subgroup analysis) for the included studies. **Figure S10.** TSA of mortality at the longest follow-up (subgroup analysis) for the included studies. **Figure S11.** Secondary outcomes for the included studies. **Figure S12.** Funnel plot of secondary outcomes for the included studies. **Figure S13.** TSA of secondary outcomes for the included studies. **Figure S14.** TSA of adverse evevts for the included studies. **Figure S15.** TSA of adverse evevts (subgroup analysis) for the included studies. **Table S5.** Meta-analysis of different oxygenation targets in recent years. **Table S6.** PRISMA2020 checklist.

## Data Availability

Not applicable.

## Abbreviations

COT: Conservative oxygen therapy
LOT: Liberal oxygen therapy
ICU: Intensive care unit
RCT: Randomized controlled trial
MVF: Days free from mechanical ventilation support
VFT: Vasopressor-free time
FiO_2_: Fraction of inspired oxygen
PaO_2_: Arterial partial pressure of oxygen
SaO_2_: Arterial oxygen saturation
SpO_2_: Peripheral oxygen saturation
P/F: PaO_2_/FiO_2_ ratios
RR: Risk ratios
CIs: 95% Confidence intervals
MDs: Mean differences
SD: Standard deviation
IQRs: Interquartile ranges
TSA: Trial sequential analysis
RRR: Relative risk reduction
GRADE: Grading of Recommendations Assessment, Development and Evaluation
