# Supplementary material for: Conservative versus liberal oxygen therapy for intensive care unit patients: meta-analysis of randomized controlled trials
Source: Ann Intensive Care. 2024 Apr 26;14:68. doi: 10.1186/s13613-024-01300-7 (PMC11052962; doi:10.1186/s13613-024-01300-7)
Supplement: Supplementary file 1 — Additional file 1: Search strategy. Table S1. Demographic details of the included studies. Table S2. Primary diagnosis or comorbidities of the included studies. Table S3. Comorbidities of the included studies in medical ICU and Mixed ICU. Table S4. Adverse events of the included studies. Figure S1. Assessment on the risk of bias for the included RCTs. Figure S2. Funnel plot of mortality at the longest follow-up for the included studies. Figure S3. The GRADE assessment of the certainty of the evidence. Figure S4. Mortality at 30 days and TSA of the the included studies. Figure S5. Mortality at 90 days and TSA of the the included studies. Figure S6. Subgroup analysis of 30-day mortality for the included studies. Figure S7. Subgroup analysis of 90-day mortality for the included studies. Figure S8. TSA of 30-day mortality (subgroup analysis) for the included studies. Figure S9. TSA of 90-day mortality (subgroup analysis) for the included studies. Figure S10. TSA of mortality at the longest follow-up (subgroup analysis) for the included studies. Figure S11. Secondary outcomes for the included studies. Figure S12. Funnel plot of secondary outcomes for the included studies. Figure S13. TSA of secondary outcomes for the included studies. Figure S14. TSA of adverse evevts for the included studies. Figure S15. TSA of adverse evevts (subgroup analysis) for the included studies. Table S5. Meta-analysis of different oxygenation targets in recent years. Table S6. PRISMA2020 checklist. [file 13613_2024_1300_MOESM1_ESM.docx]

**Conservative versus liberal oxygen therapy for intensive care unit patients: Meta-analysis of randomized controlled trials**

| **Results** |  |
| --- | --- |
| 1. Search strategy | p 2 |
| 1. Table S1. Demographic details of the included studies. | p 3-4 |
| 1. Table S2. Primary diagnosis or comorbidities of the included studies. | p 5-8 |
| 1. Table S3. Comorbidities of the included studies in medical ICU and Mixed ICU. | p 9 |
| 1. Table S4. Adverse events of the included studies. | p 10-11 |
| 1. Figure S1. Assessment on the risk of bias for the included RCTs. | p 12 |
| 1. Figure S2. Funnel plot of mortality at the longest follow-up for the included studies. | p 13 |
| 1. Figure S3. The GRADE assessment of the certainty of the evidence | p 14 |
| 1. Figure S4. Mortality at 30 days and TSA of the the included studies. | p 15 |
| 1. Figure S5. Mortality at 90 days and TSA of the the included studies. | p 16 |
| 1. Figure S6. Subgroup analysis of 30-day mortality for the included studies. | p 17 |
| 1. Figure S7. Subgroup analysis of 90-day mortality for the included studies. | p 18 |
| 1. Figure S8. TSA of 30-day mortality (subgroup analysis) for the included studies. | p 19 |
| 1. Figure S9. TSA of 90-day mortality (subgroup analysis) for the included studies. | p 20 |
| 1. Figure S10. TSA of mortality at the longest follow-up (subgroup analysis) for the included studies. | p 21 |
| 1. Figure S11. Secondary outcomes for the included studies. | p 22 |
| 1. Figure S12. Funnel plot of secondary outcomes for the included studies. | p 23 |
| 1. Figure S13. TSA of secondary outcomes for the included studies. | p 24 |
| 1. Figure S14. TSA of adverse evevts for the included studies. | p 25 |
| 1. Figure S15. TSA of adverse evevts (subgroup analysis) for the included studies. | p 26 |
| 1. Table S5. Meta-analysis of different oxygenation targets in recent years. | p 27-30 |
| 1. Table S6. PRISMA2020 checklist | P 31-34 |

1. **Search strategy**

**((((((((((((((('conventional oxygen'[Title/Abstract]) OR ('liberal oxygen'[Title/Abstract])) OR ('conservative oxygen'[Title/Abstract])) OR (hyperoxia[Title/Abstract])) OR (hyperoxemia[Title/Abstract])) OR ('oxygen therapy'[Title/Abstract])) OR ('arterial oxygen saturation'[Title/Abstract])) OR ('high* oxygen'[Title/Abstract])) OR ('low* oxygen'[Title/Abstract])) OR ('oxygen* target'[Title/Abstract])) OR ('oxygen strategies'[Title/Abstract])) OR ('blood gas'[Title/Abstract])) OR (pao2[Title/Abstract] OR sao2[Title/Abstract] OR fio2[Title/Abstract] OR spo2[Title/Abstract])) OR (hyperoxia[MeSH Terms])) OR ((inspir*[Title/Abstract] OR inhal*[Title/Abstract] OR fraction*[Title/Abstract] OR concentrat*[Title/Abstract] OR arterial*[Title/Abstract] OR saturation[Title/Abstract] OR level*[Title/Abstract] OR tension*[Title/Abstract] OR supply*[Title/Abstract] OR supplement*[Title/Abstract] OR supplie*[Title/Abstract] OR therap*[Title/Abstract] OR administr*[Title/Abstract] OR dosag*[Title/Abstract] OR dose*[Title/Abstract] OR dosing*) oxygen[Title/Abstract])) OR (oxygen therapy[MeSH Terms]) OR (hyperoxia[MeSH Terms])** AND **((((((((((((((adult*[MeSH Major Topic]) OR (adult*[Title/Abstract])) OR (age*[Title/Abstract])) OR (18 years old[Title/Abstract])) OR (18yrs[Title/Abstract])) OR (older*[Title/Abstract])) OR (elder*[Title/Abstract])) NOT (child*[Title/Abstract])) NOT (infant*[Title/Abstract])) NOT (newborn*[Title/Abstract])) NOT (animal*[MeSH Terms])) NOT (experiment[Title/Abstract])) NOT (premature[Title/Abstract])) NOT (pregnan*[Title/Abstract])) NOT (pediatric*[Title/Abstract])** AND **((((((((((('clinical trials'[Title/Abstract]) OR (random*[Title/Abstract])) OR ('single blind'[Title/Abstract])) OR ('double blind'[Title/Abstract])) OR ('random* control* trial'[Title/Abstract])) OR ('random* control* trial'[MeSH Terms])) OR (crossover[Title/Abstract])) OR (placebo[Title/Abstract])) OR (prospective[Title/Abstract])) OR ('random allocation'[Title/Abstract])) OR (RCT[Title/Abstract])) OR ('random* controlled'[Title/Abstract])**

Table S1. Demographic details of the included studies.

| Study | Study period | Population^θ^ | | Demographics,Mean (SD) | | | | | | APACHE  Median (IQR) | | SOFA  Mean (SD) | | Type of ICU admission, n (%) | | | | | |
| --- | --- | --- | --- | --- | --- | --- | --- | --- | --- | --- | --- | --- | --- | --- | --- | --- | --- | --- | --- |
|  |  |  |  | Age (years) | | Sex (Male), n (%) | | BMI (Kg/m^2^) | |  |  |  |  | Medical | | Surgical | | Others | |
|  |  | COT | LOT | COT | LOT | COT | LOT | COT | LOT | COT | LOT | COT | LOT | COT | LOT | COT | LOT | COT | LOT |
| Asfar  2017 | 2012.11.3-  2014.6.13 | 217 | 217 | 66.3  (14.6) | 67.8  (12.7) | 140 (65) | 137 (63) | ND | ND | ND | ND | 10.3 (2.9)^&^ | 10.2  (2.7)^&^ | ND | ND | ND | ND | ND | ND |
| Barrot 2020 | 2016.6- 2018.9 | 99 | 102 | 63.0  (15.5) | 63.5 (14.5) | 65 (65.7) | 64 (62.7) | 27.9  (7.2) | 27.9  (6.6) | ND | ND | 9.3  (3.7)^&^ | 8.9  (3.6)^&^ | ND | ND | ND | ND | ND | ND |
| Gelissen 2021 | 2015.2- 2018.10 | 205 | 195 | 66.7  (14.9) | 68 (10.5) | 134 (65) | 126 (65) | ND | ND | ND | ND | 5.3 (3.7)^@^ | 6 (3.0)^@^ | 143 (70) | 139 (71) | 50 (24) | 41 (21) | 12 (6) | 15 (8) |
| Girardis 2016 | 2010.3.1-  2012.10.30 | 216 | 218 | 62.7  (17.2) | 64.3  (8.2) | 121  (56.0) | 125 (57.3) | ND | ND | ND | ND | ND | ND | 77 (35.7) | 86 (39.5) | 139  (64.3) | 132  (60.7) | 0 | 0 |
| Klitgaard 2022^∆^ | 2017.6.20- 2020.8.3 | 82 | 86 | 66.7  (10.6) | 68.3  (6.8) | 54  (66) | 64  (74) | ND | ND | ND | ND | 9.7 (3.0)^§^ | 8 (4.5)^§^ | 82 (100) | 86 (100) | 0 | 0 | 0 | 0 |
| Mackle 2020 | 2015.9- 2018.5 | 484 | 481 | 58.1  (16.2 ) | 57.5  (16.1) | 306  (63.2) | 302  (62.8) | ND | ND | 23.6 ± 9.3^#^ | 23.3 ± 9.4^#^ | ND | ND | 335  (69.2) | 335  (69.6) | 149  (30.8) | 146  (30.4) | 0 | 0 |
| Martin 2021 | 2018.1-  2019.4 | 17 | 17 | 66 (12.4) | | 22 (64.7) | | ND | | 23 (17–29)^#^ | | 11 (9–13)^&^ | | 17 (100) | 17 (100) | 0 | 0 | 0 | 0 |
| Nafae  2023 | 2018.7-  2021.1 | 28 | 28 | 52.8  (8.9) | 48.9  (9.8) | 19 (67.9) | 17 (60.7) | ND | ND | ND | ND | ND | ND | 28 (100) | 28 (100) | 0 | 0 | 0 | 0 |
| Nielsen 2024 | 2020.8.25- 2023.3.8 | 351 | 346 | 64  (14.1) | 66.3 (14.1) | 253 (69.9) | 242 (67.6) | ND | ND | ND | ND | 3.3  (2.2)^§^ | 3.7  (3.0)^§^ | 351 (100) | 346 (100) | 0 | 0 | 0 | 0 |
| Panwar 2016 | 2013.6-  2014.10 | 52 | 51 | 62.4  (14.9) | 62.4  (17.4) | 32 (62) | 33 (65) | 27.6 (10.3) | 27.6 (10.1) | 79.5 (61-92.5)* | 70  (50-84)* | 7.9 (2.9)^&^ | 7.4 (3.1)^&^ | 39 (75) | 41 (80) | 10 (19) | 8 (16) | 3 (6) | 2 (4) |
| Rasmussen 2021^∆^ | 2017.6.20- 2020.8.3 | 54 | 55 | 69 (12.2) | 68 (11.4) | 43 (79.6) | 43 (76.8) | ND | ND | ND | ND | 6 (3.0)^§^ | 6 (3.0)^§^ | 54 (100) | 55 (100) | 0 | 0 | 0 | 0 |
| Schjørring 2021 | 2017.6.20- 2020.8.3 | 1453 | 1457 | 69  (12.6) | 69  (12.6) | 925 (63.7) | 946 (64.9) | ND | ND | ND | ND | 7.7 (3.7)^§^ | 7.7 (3.7)^§^ | 1248 (85.9) | 1240 (85.1) | 18  (1.2) | 21  (1.4) | 187  (12.9) | 196 (12.9) |
| Semler 2022 | 2018.7.1- 2021.8.31 | 808^a^, 859^b^ | 874^c^ | 56(17.1)^a^  58(15.6)^b^ | 57.3  (17.1)^c^ | 447(55.3)^a^  474(55.2)^b^ | 465  (53.2)^c^ | ND | ND | ND | ND | 5.7 (3.0)^a^  5.7 (3.0)^b^ | 5.3  (3.7)^c^ | 528(65.3)^a^  546(63.6)^b^ | 592 (67.7)^c^ | ND | ND | 280(34.7)^a^313(36.4)^b^ | 282  (32.3)^c^ |
| van der Wal 2023 | 2018.11- 2021.11 | 335 | 329 | 66.7  (11.2) | 65.3 (12.7) | 111  (33.1) | 118  (35.9) | ND | ND | 87 (66-107)^^^ | 86  (65-113)^^^ | ND | ND | 258  (77.2) | 251  (76.3) | 76  (22.8) | 78  (23.7) | ND | ND |
| Yang  2019 | 2017.2.17-2017.10.24 | 100 | 114 | 57  (17.3) | 58.3  (17.3) | 69  (69) | 69  (60.5) | ND | ND | 17  (12–21)^#^ | 17 (12–22)^#^ | ND | ND | 88 (88) | 94 (82.5) | 12  (12) | 14  (12.3) | ND | ND |

Abbreviations: BMI: body mass index; COT: conservative oxygen therapy; LOT:liberal oxygen therapy; ND: no data.

^Θ^: patients included in primary analysis;^#^APACHE II:Acute Physiology and Chronic Health Evaluation score II is the sum of three components at the first 24 hours after admission to the ICU: an acute physiology score (0 to 60), chronic health evaluation score (0 to 5), and age score (0 to 6), with total score ranging from 0 to 71, and higher scores indicating more severe disease and a higher risk of death; *APACHE III: Acute Physiology and Chronic Health Evaluation score III is the sum of three components at the time of randomization: an acute physiology score (0 to 252), chronic health evaluation score (0 to 23), and age score (0 to 24), with total score ranging from 0 to 299, and higher scores indicating more severe disease and a higher risk of death; ^APACHE IV: an acute physiology score (0 to 252), age score (0 to 6), chronic health status score (0 to 23), previous Health status, admission diagnosis, ICU admission source, ICU type; ^&^SOFA: Sequential Organ Failure Assessment score includes subscores ranging from 0-4 for each of five organ system (circulation, lungs, liver, kidneys and coagulation), with score ranging from 0-20, and higher scores indicating more severe organ failure; ^§^ SOFA: range from 0 to 24, with higher scores indicating more severe organ failure; ^@^The SOFA score is a cumulativescore of the respiratory system(PaO_2_/FiO_2_), nervous system(Glasgow Coma Scale),cardiovascular system (meanarterial pressure or vasopressoruse), liver (bilirubin), coagulation(platelets), and kidney function(creatinine or urine output) used toassess the severity of organ failurein the ICU. Each system scores 0 to 4 for a total of 0 to 24. If therespiratory system is excluded, themaximum score is 20; a:in a lower SpO_2_ target (90%; goal range, 88 to 92%); b:in an intermediate SpO_2_ target (94%; goal range, 92 to 96%); c:in a higher SpO_2_ target (98%; goal range, 96 to 100%); ^∆^ Substudy of HOT-ICU (Schjørring2021^39^).

Table S2. Primary diagnosis or comorbidities of the included studies, n (%).

| Study | Respiratory system | | | | | | | | | | Circulatory system | | | | | | | |
| --- | --- | --- | --- | --- | --- | --- | --- | --- | --- | --- | --- | --- | --- | --- | --- | --- | --- | --- |
|  | Respiratory failure | | COPD | | ARDS | | Pneumonia | | Others | | Ischemic heart disease | | Heart failure | | CAD | | Cardiac arrest | |
|  | COT | LOT | COT | LOT | COT | LOT | COT | LOT | COT | LOT | COT | LOT | COT | LOT | COT | LOT | COT | LOT |
| Asfar 2017 | ND | ND | 31 (14) | 35 (16) | ND | ND | ND | ND | ND | ND | ND | ND | 13 (6) | 11 (5) | 25 (12) | 26 (12) | ND | ND |
| Barrot 2020 | ND | ND | ND | ND | 99 (100) | 102 (100) | ND | ND | 20 (20) | 28 (27) | ND | ND | 5 (5) | 9 (9) | 6 (6.1) | 7 (7) | ND | ND |
| Gelissen 2021 | ND | ND | 15 (7) | 20 (10) | ND | ND | 69 (34) | 59 (30) | ND | ND | ND | ND | 4(2) | 3 (2) | 10 (5) | 12 (6) | 37(18) | 42 (22) |
| Girardis 2016 | 121 (56) | 129 (59) | 7 (3) | 11 (5) | ND | ND | ND | ND | ND | ND | ND | ND | ND | ND | ND | ND | ND | ND |
| Klitgaard 2022 | ND | ND | 8 (10) | 8 (9) | 14 (17) | 15 (17) | 49 (60) | 54(63) | ND | ND | 4 (5) | 9 (11) | 3 (4) | 8 (9) | ND | ND | 9 (11) | 6 (7) |
| Mackle 2020 | 15 (3) | 9 (2) | ND | ND | ND | ND | ND | ND | ND | ND | ND | ND | ND | ND | 15 (3) | 7 (2) | ND | ND |
| Martin 2021 | ND | ND | ND | ND | ND | ND | 27 (79) | | 7 (21) | | 19 (56) | | | | | | | |
| Nafae 2023 | ND | ND | ND | ND | ND | ND | 20 (71) | 17 (61) | 8 (29) | 11(40) | ND | ND | ND | ND | ND | ND | ND | ND |
| Nielsen 2024 | ND | ND | 28 (8) | 24 (7) | 149 (41) | 144 (40) | 265 (73) | 258(72) | ND | ND | 38 (11) | 34 (10) | ND | ND | ND | ND | 2 (0.6) | 5 (2) |
| Panwar 2016 | ND | ND | 11(21) | 5 (10) | 17 (33) | 10 (20) | ND | ND | ND | ND | 6 (12) | 5 (10) | ND | ND | ND | ND | ND | ND |
| Rasmussen2021 | ND | ND | 6 (11) | 5 (9) | ND | ND | 54 (100) | 56 (100) | ND | ND | 6 (11) | 6 (11) | 2 (4) | 3 (5) | ND | ND | ND | ND |
| Schjørring  2021 | ND | ND | 277  (19) | 286 (20) | 178 (12) | 195 (13) | 838 (58) | 836 (57) | ND | ND | 205 (14) | 205(14) | 140  (10) | 146 (10) | 84 (6) | 99 (7) | 149  (10) | 186 (13) |
| Semler 2022 | ND | ND | 148 (18)^a^ 175 (20)^b^ | 169  (19)^c^ | ND | ND | ND | ND | ND | ND | 136 (17)^a^  138 (16)^b^ | 145  (17)^c^ | ND | ND | 145 (18)^a^  152 (18)^b^ | 178 (20)^c^ | 125 (16)^a^  100 (12)^b^ | 109 (13)^c^ |
| van der Wal 2023 | ND | ND | 39 (12) | 37 (11) | ND | ND | 54 (16) | 43 (13) | 6 (2) | 1 (0.3) | ND | ND | 2 (0.6) | 9 (3) | ND | ND | 89 (27) | 96 (29) |
| Yang 2019 | 63 (63) | 53 (47) | ND | ND | ND | ND | ND | ND | ND | ND | ND | ND | ND | ND | ND | ND | ND | ND |

Table S2. Primary diagnosis or comorbidities of the included studies, n (%) (Continued).

| Study | Digestive system | | | | | | | | Shock^#^ | | Renal system | | | |
| --- | --- | --- | --- | --- | --- | --- | --- | --- | --- | --- | --- | --- | --- | --- |
|  | Liver failure | | Intestinal ischemia | | Cirrhosis | | Others | | Shock | | Renal failure | | CRRT therapy | |
|  | COT | LOT | COT | LOT | COT | LOT | COT | LOT | COT | LOT | COT | LOT | COT | LOT |
| Asfar 2017 | ND | ND | ND | ND | 12(6) | 8 (4) | ND | ND | ND | ND | 23 (11) | 23 (11) | ND | ND |
| Barrot 2020 | ND | ND | ND | ND | 6 (6) | 6 (6) | ND | ND | ND | ND | 10 (10) | 8 (8) | ND | ND |
| Gelissen 2021 | ND | ND | ND | ND | 3 (2) | 4 (2) | ND | ND | ND | ND | 17 (8) | 18 (9) | 6 (3) | 7 (4) |
| Girardis 2016 | 40 (19) | 45 (21) | ND | ND | ND | ND | 28 (13) | 31 (14) | 68 (31) | 72 (33) | 13 (6) | 13 (6) | ND | ND |
| Klitgaard 2022 | ND | ND | 2 (2) | 3 (4) | ND | ND | ND | ND | ND | ND | ND | ND | 1 (1) | 1(1) |
| Mackle 2020 | ND | ND | ND | ND | ND | ND | 16 (3) | 8 (2) | ND | ND | 14 (3) | 10 (2) | ND | ND |
| Martin 2021 | 7 (21) | | | | | | | | ND | ND | 3 (9) | | | |
| Nafae 2023 | ND | ND | ND | ND | ND | ND | ND | ND | ND | ND | ND | ND | ND | ND |
| Nielsen 2024 | ND | ND | 2 (0.6) | 0 | ND | ND | ND | ND | ND | ND | ND | ND | 9 (3) | 8 (2) |
| Panwar 2016 | ND | ND | ND | ND | ND | ND | ND | ND | ND | ND | ND | ND | ND | ND |
| Rasmussen 2021 | ND | ND | ND | ND | ND | ND | ND | ND | ND | ND | ND | ND | 2 (4) | 0 |
| Schjørring 2021 | ND | ND | 27 (2) | 41 (3) | ND | ND | ND | ND | ND | ND | ND | ND | 19 (1) | 28 (2) |
| Semler 2022 | ND | ND | ND | ND | ND | ND | ND | ND | 275 (34)^a^ 247 (29)^b^ | 283 (32)^c^ | 231 (31)^a^  248 (31)^b^ | 243 (29)^c^ | 52 (6)^a^  46 (5)^b^ | 39 (5)^c^ |
| van der Wal 2023 | ND | ND | ND | ND | 14 (4) | 14 (4) | ND | ND | 53 (16) | 42 (13) | 20 (6) | 22 (7) | 6 (2) | 3 (0.9) |
| Yang 2019 | ND | ND | ND | ND | ND | ND | ND | ND | 27 (27) | 29 (25) | 14 (14) | 17 (15) | 59 (59) | 65 (57) |

| Study  Table S2. Primary diagnosis or comorbidities of the included studies, n (%) (Continued). | Neurological system | | | | Endocrine system | | Hematological system | | Cancer | | Infection | |
| --- | --- | --- | --- | --- | --- | --- | --- | --- | --- | --- | --- | --- |
|  | Stroke | | Others | | Diabetes | |  |  |  |  |  |  |
|  | COT | LOT | COT | LOT | COT | LOT | COT | LOT | COT | LOT | COT | LOT |
| Asfar 2017 | ND | ND | ND | ND | ND | ND | ND | ND | 68 (31) | 62 (29) | ND | ND |
| Barrot 2020 | ND | ND | ND | ND | 23 (23) | 21 (21) | ND | ND | 24 (24) | 20 (20) | ND | ND |
| Gelissen 2021 | 5 (2) | 6 (3) | ND | ND | 41 (20) | 43 (22) | 10 (5) | 10 (5) | 17 (8) | 15 (8) | 71 (35) | 73 (38) |
| Girardis 2016 | ND | ND | ND | ND | ND | ND | ND | ND | 72 (33.3) | 70 (31.1) | 81 (38) | 88 (40) |
| Klitgaard 2022 | 1 (1) | 1(1) | ND | ND | ND | ND | ND | ND | 0 | 1 (1) | ND | ND |
| Mackle 2020 | ND | ND | ND | ND | ND | ND | ND | ND | 10 (2) | 8 (2) | ND | ND |
| Martin 2021 | ND | ND | ND | ND | ND | ND | ND | ND | 4 (12) | | ND | ND |
| Nafae 2023 | ND | ND | ND | ND | ND | ND | ND | ND | ND | ND | ND | ND |
| Nielsen 2024 | 1 (0.3) | 2 (0.6) | ND | ND | ND | ND | 26 (7) | 35 (10) | 8 (2) | 8 (2) | ND | ND |
| Panwar 2016 | ND | ND | ND | ND | ND | ND | ND | ND | ND | ND | ND | ND |
| Rasmussen 2021 | ND | ND | ND | ND | ND | ND | 5 (9) | 3 (5) | 1(2) | 2 (4) | ND | ND |
| Schjørring 2021 | 25 (2) | 22 (2) | 9 (0.6) | 15 (1) | ND | ND | 82 (6) | 86 (6) | 65 (5) | 61(4) | ND | ND |
| Semler 2022 | ND | ND | ND | ND | ND | ND | ND | ND | ND | ND | ND | ND |
| van der Wal 2023 | ND | ND | 32 (10) | 32 (10) | 52 (16) | 52 (16) | 14 (4) | 19 (6) | 8 (2) | 5 (2) | ND | ND |
| Yang 2019 | ND | ND | ND | ND | ND | ND | ND | ND | ND | ND | ND | ND |

Table S2. Primary diagnosis or comorbidities of the included studies, n (%) (Continued).

| Study | Trauma | | Musculoskeletal /skin | | Immunosuppressed | | Acute abdominal infection/infarction | | Major bleeding | |
| --- | --- | --- | --- | --- | --- | --- | --- | --- | --- | --- |
|  | COT | LOT | COT | LOT | COT | LOT | COT | LOT | COT | LOT |
| Asfar 2017 | ND | ND | ND | ND | 45 (21) | 42 (19) | ND | ND | ND | ND |
| Barrot 2020 | ND | ND | ND | ND | 13 (13) | 9 (9) | ND | ND | ND | ND |
| Gelissen 2021 | ND | ND | 12 (6) | 4 (2) | 25 (12) | 22 (11) | 34 (17) | 30 (15) | 24 (12) | 17 (9) |
| Girardis 2016 | ND | ND | ND | ND | ND | ND | ND | ND | ND | ND |
| Klitgaard 2022 | ND | ND | ND | ND | ND | ND | ND | ND | ND | ND |
| Mackle 2020 | ND | ND | ND | ND | 46 (10) | 54 (11) | ND | ND | ND | ND |
| Martin 2021 | ND | ND | ND | ND | 5 (15) | | ND | ND | ND | ND |
| Nafae 2023 | ND | ND | ND | ND | ND | ND | ND | ND | ND | ND |
| Nielsen 2024 | 1 (0.3) | 0 | ND | ND | ND | ND | ND | ND | ND | ND |
| Panwar 2016 | ND | ND | ND | ND | ND | ND | ND | ND | ND | ND |
| Rasmussen 2021 | ND | ND | ND | ND | ND | ND | ND | ND | ND | ND |
| Schjørring 2021 | 24 (2) | 29 (2) | ND | ND | ND | ND | ND | ND | ND | ND |
| Semler 2022 | ND | ND | ND | ND | ND | ND | ND | ND | ND | ND |
| van der Wal 2023 | 12 (4) | 12 (4) | ND | ND | 33 (10) | 43 (13) | 29 (9) | 37 (11) | ND | ND |
| Yang 2019 | ND | ND | ND | ND | ND | ND | ND | ND | ND | ND |

Abbreviations: COPD: Chronic obstructive pulmonary disease; ARDS: Acute respiratory distress syndrome; CAD: Coronary artery disease; ^#^ Shock included septic, sepsis, hypovolemic or hemorrhagic, or Cardiogenic shock; CRRT: Continuous renal replacement therapy; COT: conservative oxygen therapy; LOT:liberal oxygen therapy; ND: no data; a:in a lower SpO_2_ target (90%; goal range, 88 to 92%); b:in an intermediate SpO_2_ target (94%; goal range, 92 to 96%); c:in a higher SpO_2_ target (98%; goal range, 96 to 100%).

Table S3. Comorbidities of the included studies in medical ICU and Mixed ICU.

|  | Mixed ICU (n /%) | Medical ICU (n /%) |
| --- | --- | --- |
| Respiratory system disease | 996/6325 (15.7%) | 634/3328 (19.1%) |
| Cardiovascular system disease | 871/6325 (13.8%) | 547/3328 (16.4%) |
| Digestive system disease | 150/6325 (2.4%) | - |
| Renal system disease | 269/6325 (4.3%） | 154/3328 (4.6%) |
| Cancer | 505/6325 (8.0%） | 16/3328 (0.5%) |
| Immunosuppression | 332/6325 (5.2%） | - |
| Shock | 56/6325(0.9%） | - |
| Endocrine System Diseases | 232/6325 (3.7%） | - |
| Hematological system | 221/6325 (3.5%) | 61/3328 (1.8%) |

Table S4. Adverse events of included studies.

| Study | Total SAE, n (%) | | Adverse events related to organ failure, n (%) | | | | | | | | | | | |
| --- | --- | --- | --- | --- | --- | --- | --- | --- | --- | --- | --- | --- | --- | --- |
|  |  |  | Total new organ failure | | Respiratory systerm | | Circulatory system | | Digestive system | | Shock | | Renal system | |
|  | COT | LOT | COT | LOT | COT | LOT | COT | LOT | COT | LOT | COT | LOT | COT | LOT |
| Asfar 2017 | 165 (76.0) | 185 (85.3) | ND | ND | 50(23.0) | 61(28.1) | 1 (0.5) | 0 | 4 (1.8) | 7 (3.2) | ND | ND | 73 (33.6) | 77 (35.5) |
| Barrot 2020 | ND | ND | ND | ND | 39 (39.4) | 33 (32.4) | 44 (44.4) | 29 (28.4) | 4 (4.0) | 4 (3.9) | ND | ND | 10 (10.1) | 10 (9.80) |
| Gelissen 2021 | 89 (43) | 79 (41) | ND | ND | 7 (3.4) | 8 (4.1) | 6 (2.9) | 7 (3.6) | 7 (3.4) | 2 (1.0) | ND | ND | 20 (10) | 21 (11) |
| Girardis 2016 | ND | ND | 41(19.0) | 56 (25.7) | 14 (6.5) | 14 (6.4) | ND | ND | 4 (1.9) | 14(6.4) | 8 (3.7) | 23(10.6) | 26 (12.0) | 21 (9.6) |
| Klitgaard 2022 | 34 (41.4) | 38 (44.2) | ND | ND | ND | ND | 1 (1.2) | 3 (3.5) | 3 (3.6) | 1 (1.2) | 31 (37.8) | 36 (41.9) | ND | ND |
| Mackle 2020 | ND | ND | ND | ND | 48 (9.9) | 56 (11.6) | ND | ND | ND | ND | ND | ND | 94 (19.4) | 108 (22.5) |
| Martin 2021^$^ | 37/75 (49.3) | 38/75(50.7) | ND | ND | 11 (29.7) | 9 (23.7) | 12 (32.4) | 9 (23.7) | 2 (5.4) | 5 (13.2) | ND | ND | 4 (10.8) | 3 (7.9) |
| Nielsen 2024 | 178 (49.2) | 187 (52.2) | ND | ND | ND | ND | 2 (0.5) | 1 (0.3) | 3 (0.8) | 1 (0.3) | 172 (47.5) | 184 (51.4) | ND | ND |
| Panwar 2016 | ND | ND | ND | ND | 11/35 (31.4)^∆^ | 11/41 (26.8)^∆^ | 9 (17.3) | 12 (23.5) | ND | ND | ND | ND | ND | ND |
| Rasmussen 2021 | 30 (55.6) | 30 (54.5) | ND | ND | ND | ND | 1(1.9) | 0 | 1(1.9) | 0 | 30 (55.6) | 29 (52.7) | ND | ND |
| Schjørring 2021 | 525 (36.1) | 555 (38.1) | ND | ND | ND | ND | 14 (1.0) | 8 (0.5) | 32 (2.2) | 29 (2.0) | 492 (33.9) | 521(35.8) | ND | ND |
| Semler 2022 | ND | ND | ND | ND | 17 (3.8)^a^  24 (4.2)^b^ | 36 (5.9)^c^ | 127 (28.4)^a^  175 (30.3)^b^ | 160 (26.3)^c^ | ND | ND | ND | ND | 112 (25.1)^a^  118 (20.4)^b^ | 97 (15.9)^c^ |
| van der Wal 2023 | 13 (3.9) | 22 (6.7) | ND | ND | ND | ND | 2 (0.6) | 7 (2.1) | 4 (1.2) | 4 (1.2) | ND | ND | ND | ND |

Table S4. Adverse events of included studies (Continued).

|  | Stroke, n (%) | | Infection, n (%) | | ICU-acquired weakness, n (%) | | Seizure, n (%) | | Delirium, n (%) | | Other adverse events, n (%) | |
| --- | --- | --- | --- | --- | --- | --- | --- | --- | --- | --- | --- | --- |
|  | COT | LOT | COT | LOT | COT | LOT | COT | LOT | COT | LOT | COT | LOT |
| Asfar 2017 | ND | ND | 45 (20.7) | 45 (20.7) | 13 (6) | 24 (11.4) | ND | ND | ND | ND | ND | ND |
| Barrot 2020 | 4 (4.0) | 1 (1.0) | 11 (11.1) | 19 (18.6) | ND | ND | 2 (2.0) | 0 | 11 (11.1) | 11 (10.8) | ND | ND |
| Gelissen 2021 | 1 (0.5) | 2 (1.0) | ND | ND | ND | ND | ND | ND | ND | ND | 147 (72) | 143 (73) |
| Girardis 2016 | ND | ND | 39 (18.1) | 50 (22.9) | ND | ND | ND | ND | ND | ND | ND | ND |
| Klitgaard 2022 | 1 (1.2) | 0 | ND | ND | ND | ND | ND | ND | ND | ND | ND | ND |
| Mackle 2020 | 0 | 1 (0.2) | ND | ND | ND | ND | ND | ND | ND | ND | 2 (0.4) | 0 |
| Martin 2021^$^ | 4 (10.8) | 6 (15.8) | ND | ND | ND | ND | ND | ND | ND | ND | 4 (10.8) | 6 (15.8) |
| Nielsen 2024 | 1 (0.3) | 1 (0.3) | ND | ND | ND | ND | ND | ND | ND | ND | ND | ND |
| Panwar 2016 | ND | ND | ND | ND | ND | ND | ND | ND | ND | ND | ND | ND |
| Rasmussen 2021 | 0 | 1 (1.8) | ND | ND | ND | ND | ND | ND | ND | ND | ND | ND |
| Schjørring 2021 | 19 (1.3) | 23 (1.6) | ND | ND | ND | ND | ND | ND | ND | ND | ND | ND |
| Semler 2022 | 12 (2.7) ^a^  17 (2.9)^b^ | 16 (2.6)^c^ | ND | ND | ND | ND | ND | ND | ND | ND | ND | ND |
| van der Wal 2023 | 4 (1.2) | 4 (1.2) | ND | ND | ND | ND | ND | ND | ND | ND | 3 (0.9) | 4 (1.2) |

Abbreviations: COT: conservative oxygen therapy; LOT:liberal oxygen therapy; ND: no data; a:in a lower SpO_2_ target (90%; goal range, 88 to 92%); b:in an intermediate SpO_2_ target (94%; goal range, 92 to 96%); c:in a higher SpO_2_ target (98%; goal range, 96 to 100%); ^$^: The number of adverse events rather than patients was reported in Martin’s study; ^∆^:New-onset ARDS was defined as subsequent occurrence of ARDS in those patients who did not have ARDS. Respiratory systerm included ARDS, respiratory failure, ventilator-associated pneumonia, pneumothorax or pneumomediastinum, hemoptysis, atelectasis, nosocomial pneumonia during ICU stay, and severe respiratory failure necessitating prone ventilation or tracheostomy; Circulatory system included myocardial ischemia, arrhythmia, new-onset atrial fibrillation, new myocardial infarction, and cardiac arrest; Digestive system included liver failure, mesenteric ischaemia, and digestive hemorrhage; Renal system included renal failure, and kidney replacement therapy.

Figure S1. Assessment on risk of bias for included RCTs.


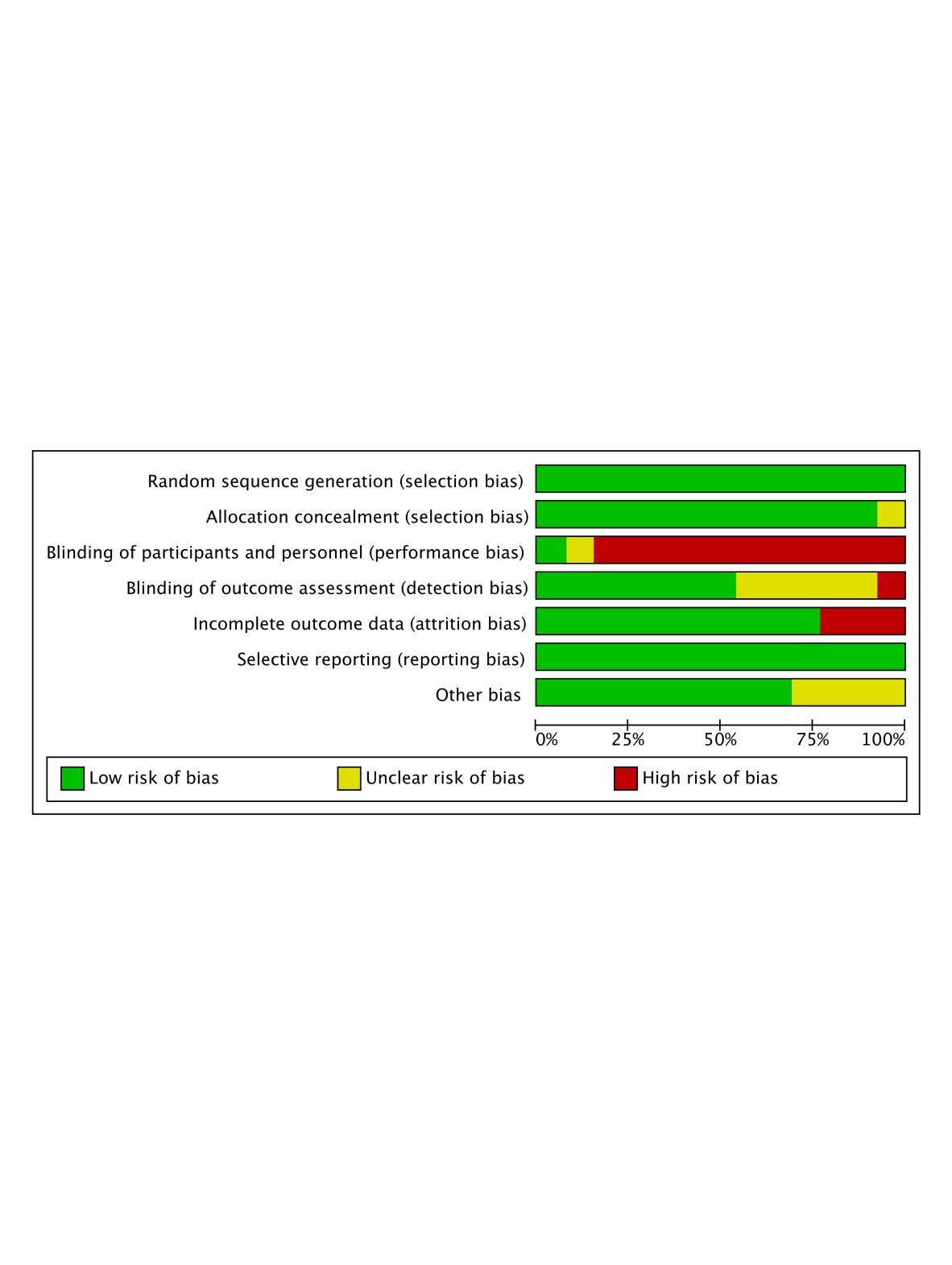

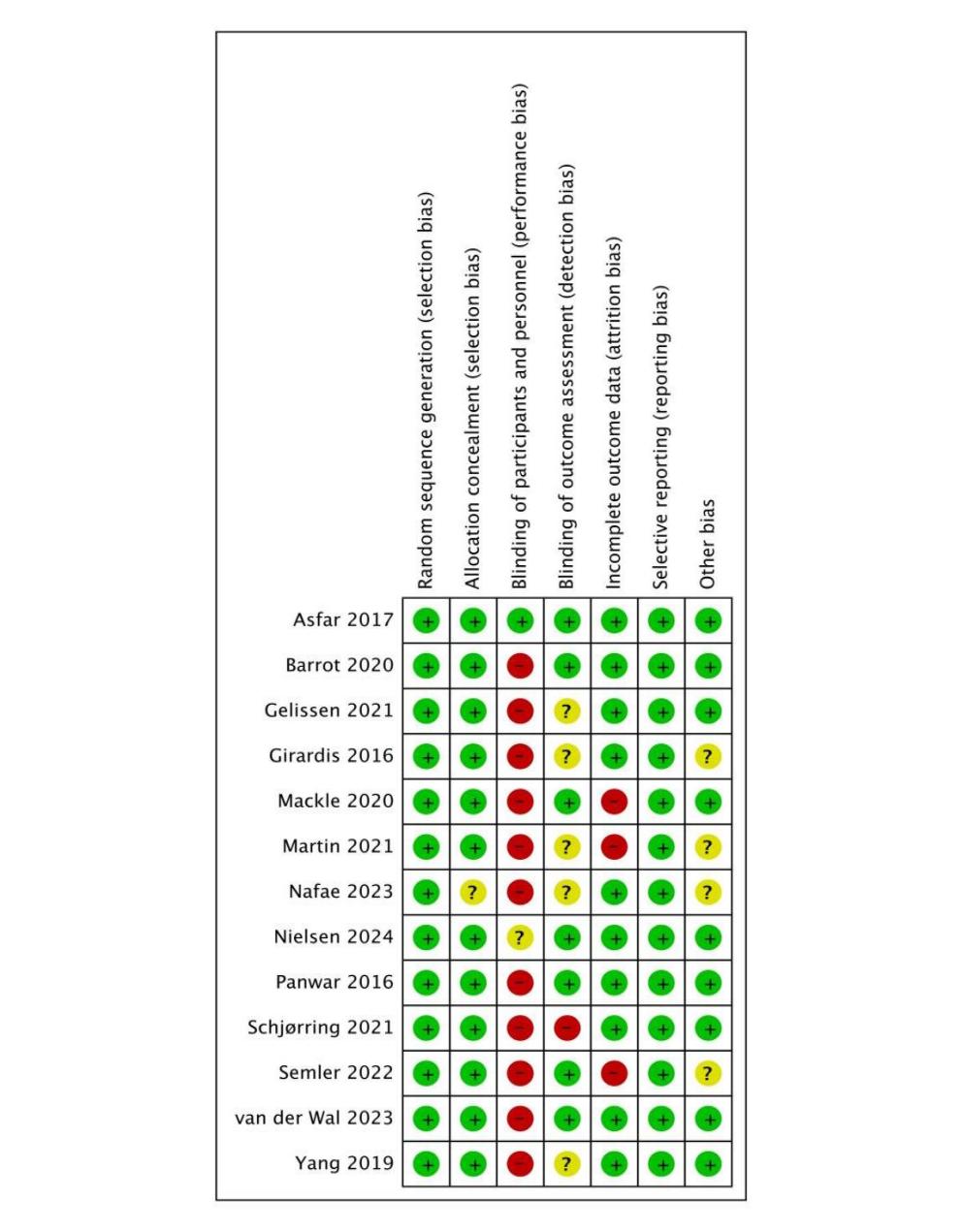


Figure S2. Funnel plot of mortality at the longest follow-up for included studies.


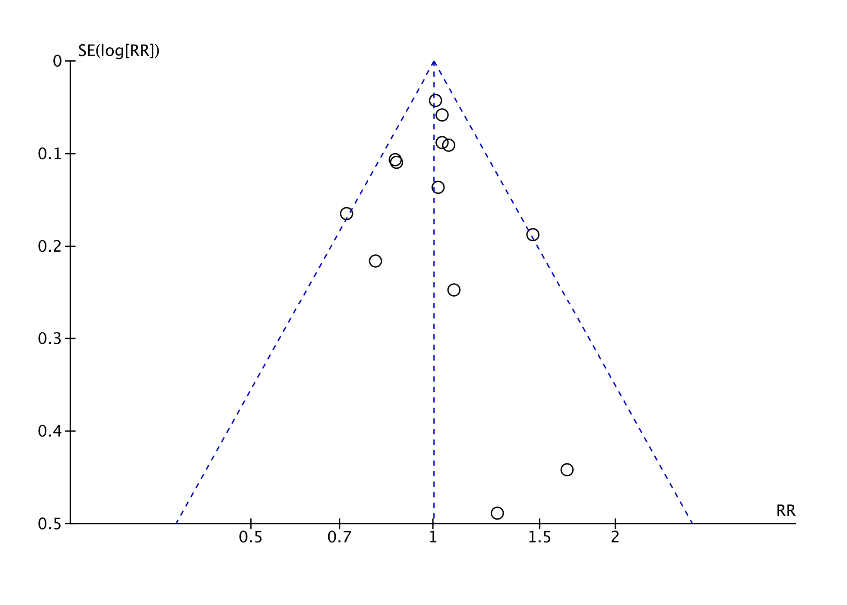


Figure S3. The GRADE of assessment of the certainty of the evidence.


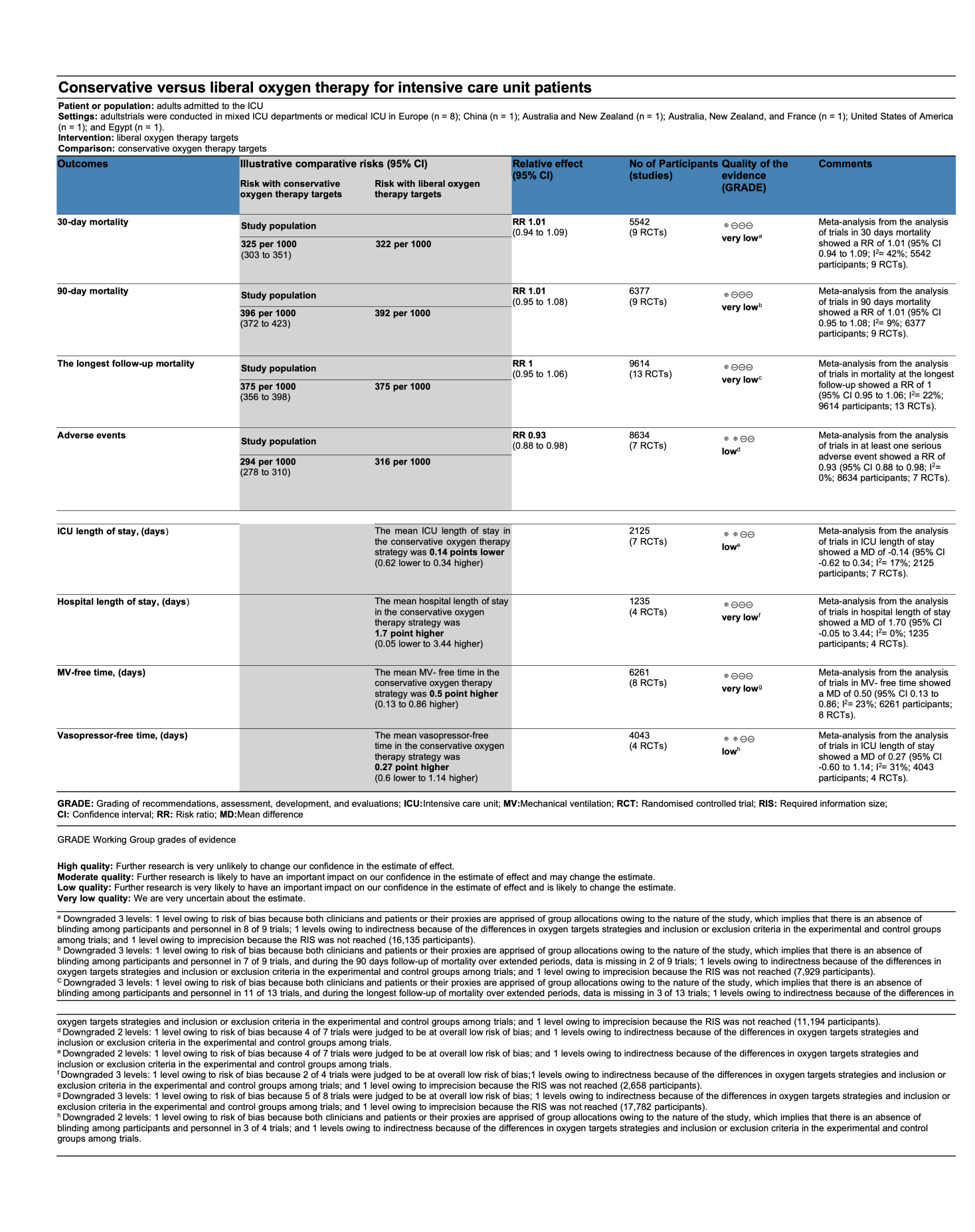


Figure S4. Mortality at 30 days and TSA of the the included studies.

**
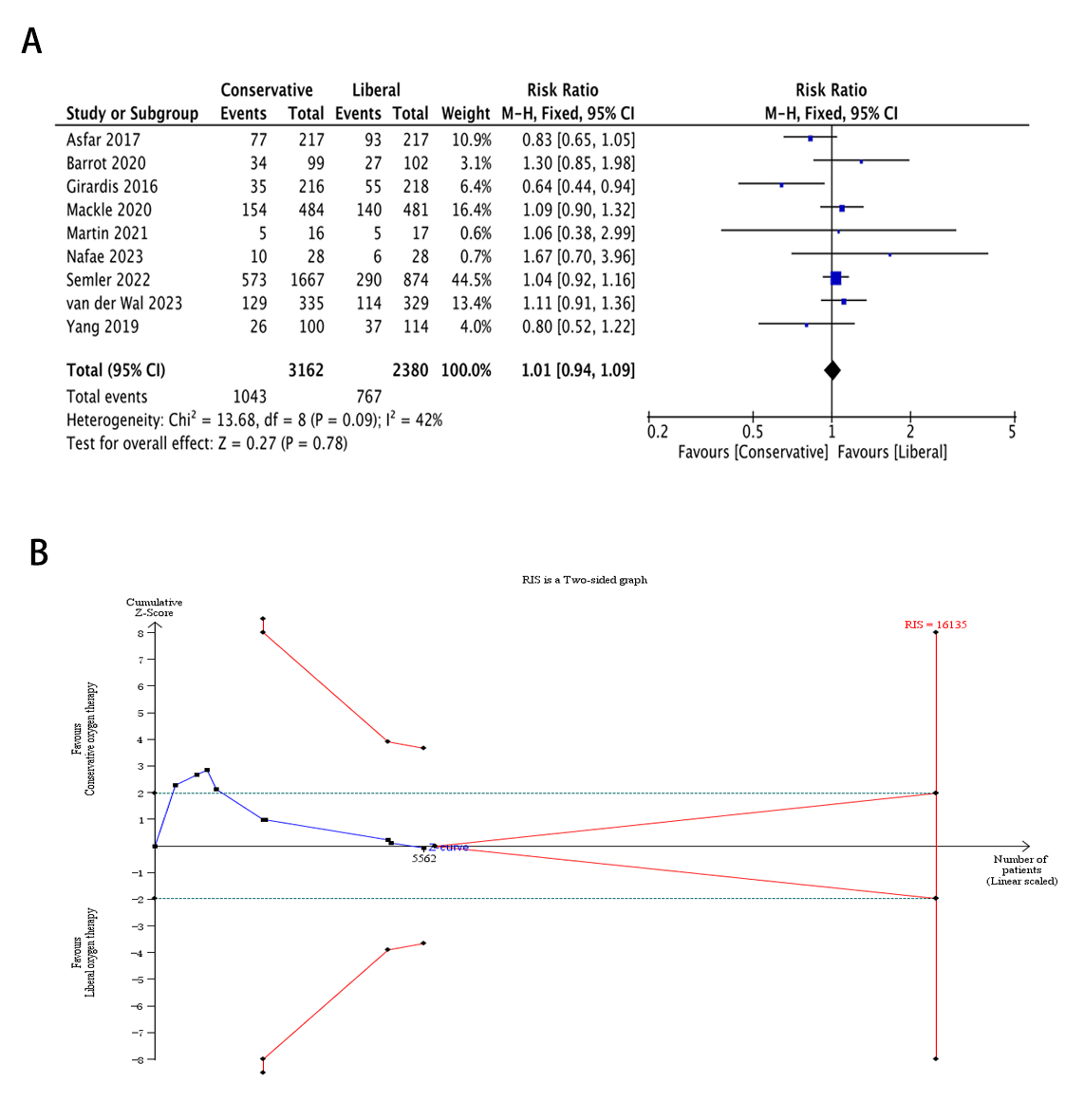
**

1. Mortality at 30 days and (B)TSA of the the included studies.

Figure S5. Mortality at 90 days and TSA of the the included studies.
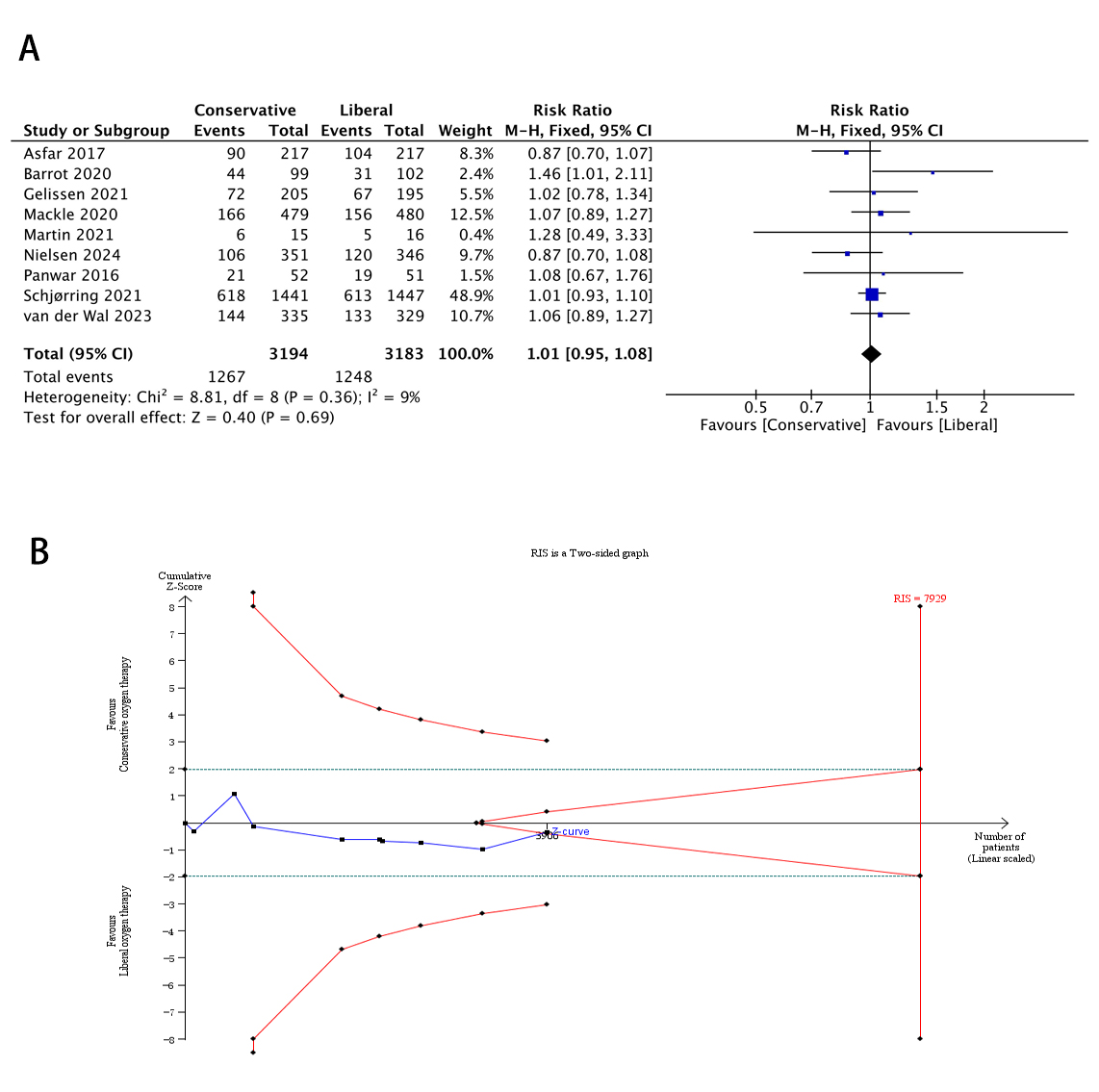


(A)Mortality at 90 days and (B)TSA of the the included studies.

Figure S6. Subgroup analysis of 30-day mortality for the included studies.
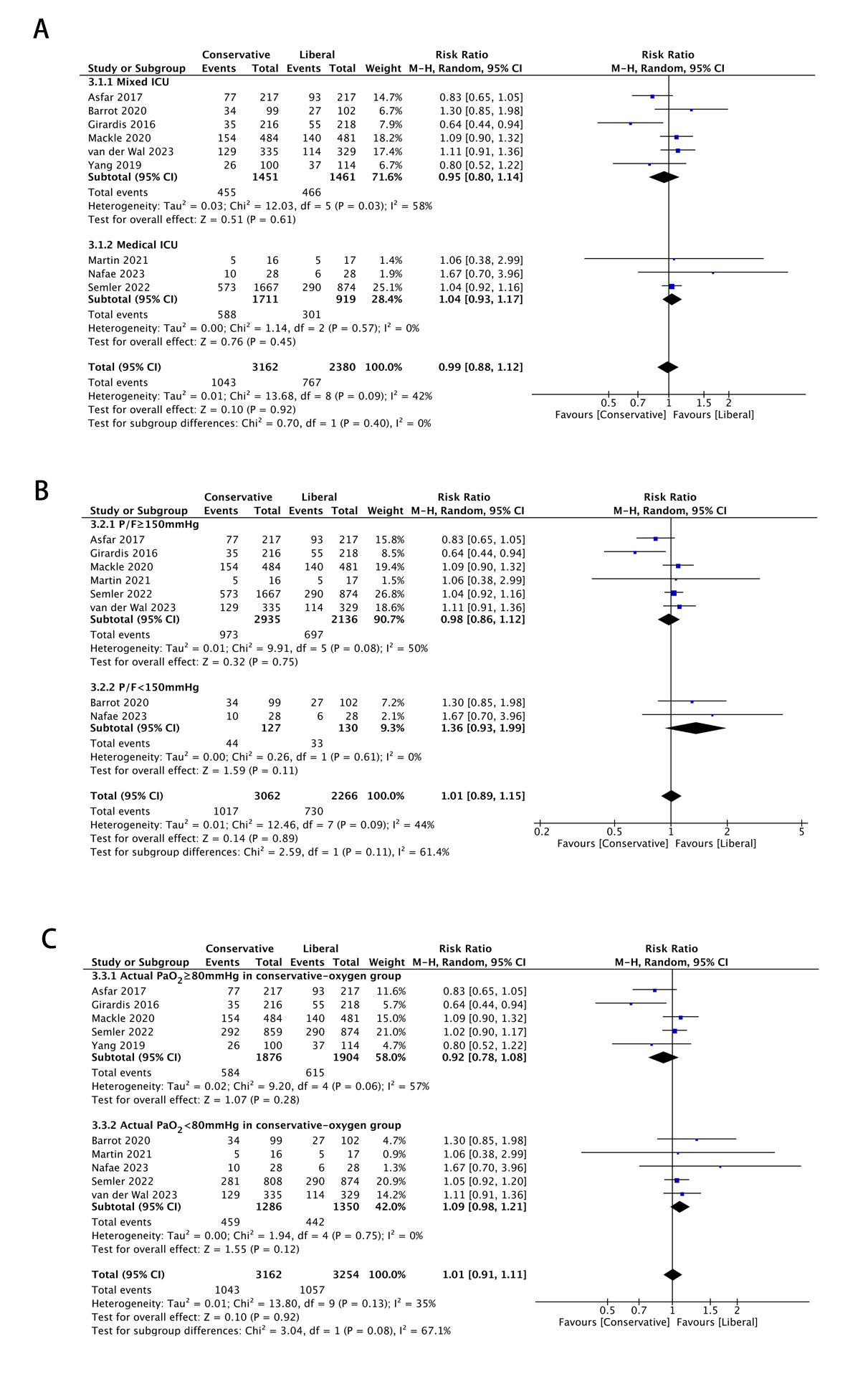


Figure S7.Subgroup analysis of 90-day mortality for the included studies.

.
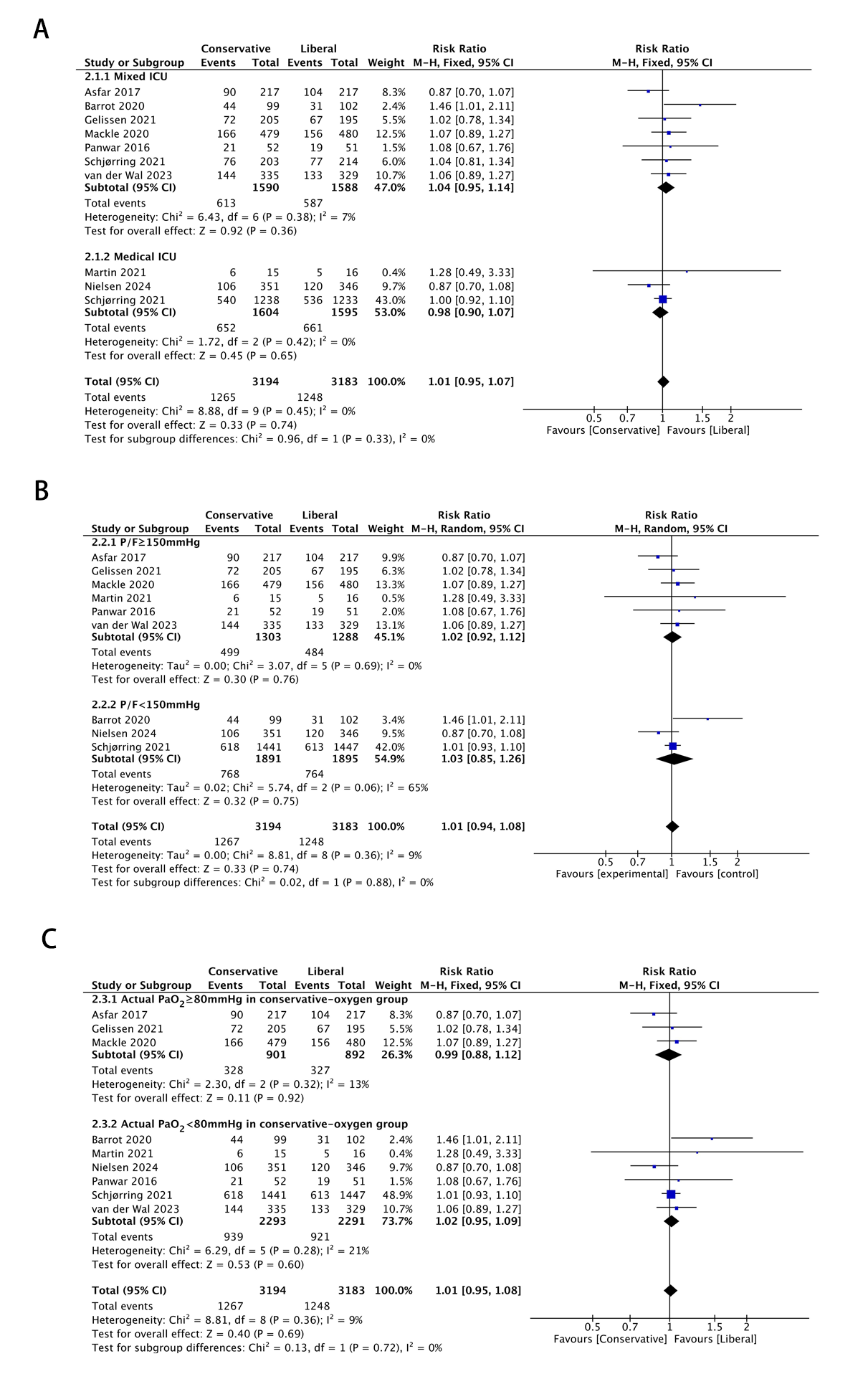


Figure S8. TSA of 30-day mortality (subgroup analysis) for included studies.

**
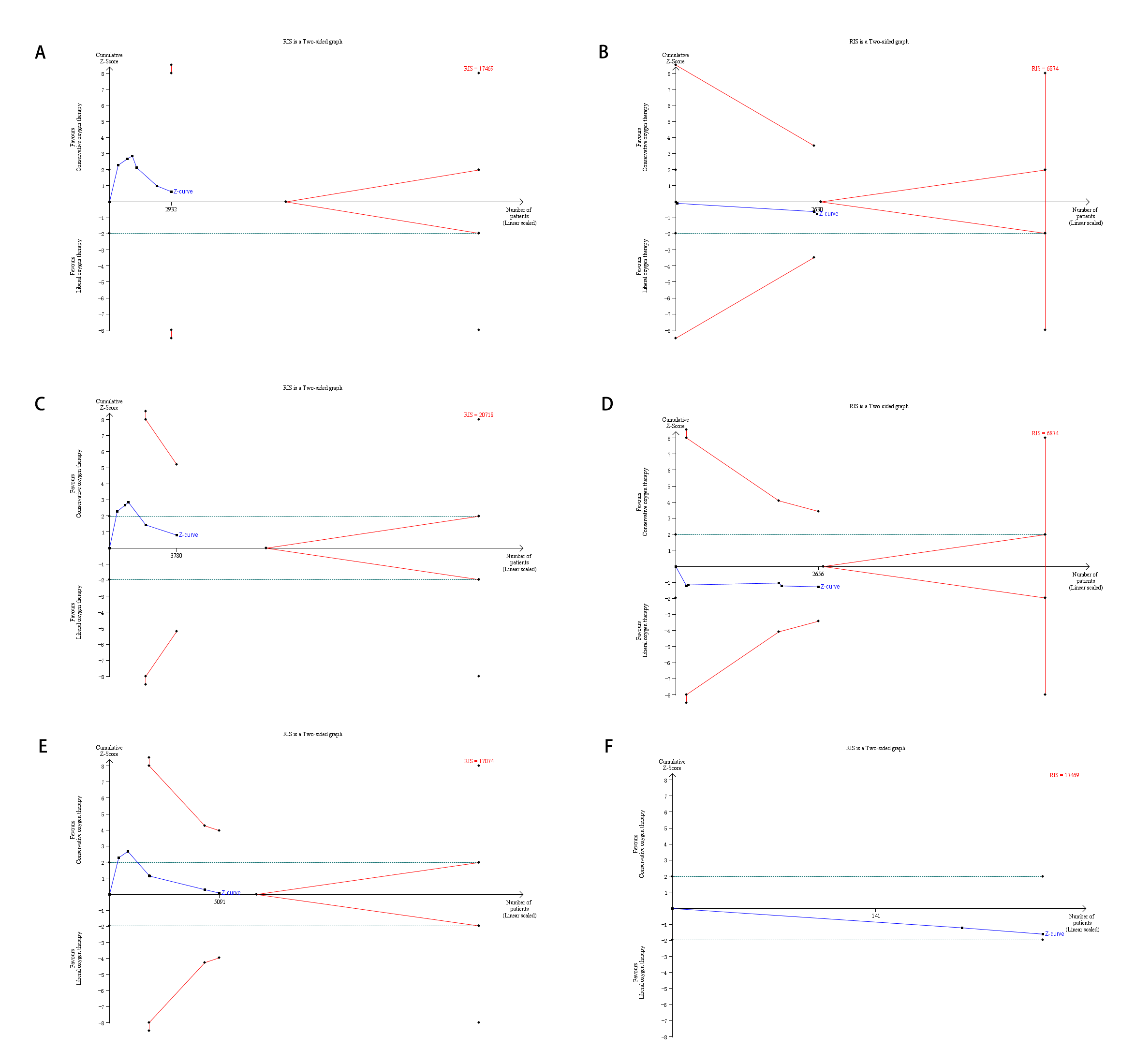
**

(A) Mixed ICU, (B) Medical ICU, (C) baseline P/F ≥ 150 mmHg, (D) baseline P/F < 150 mmHg, (E) actual PaO_2_ ≥80 mmHg in COT group, and (F) actual PaO_2_ <80 mmHg in COT group.

Figure S9. TSA of 90-day mortality (subgroup analysis) for included studies.

**
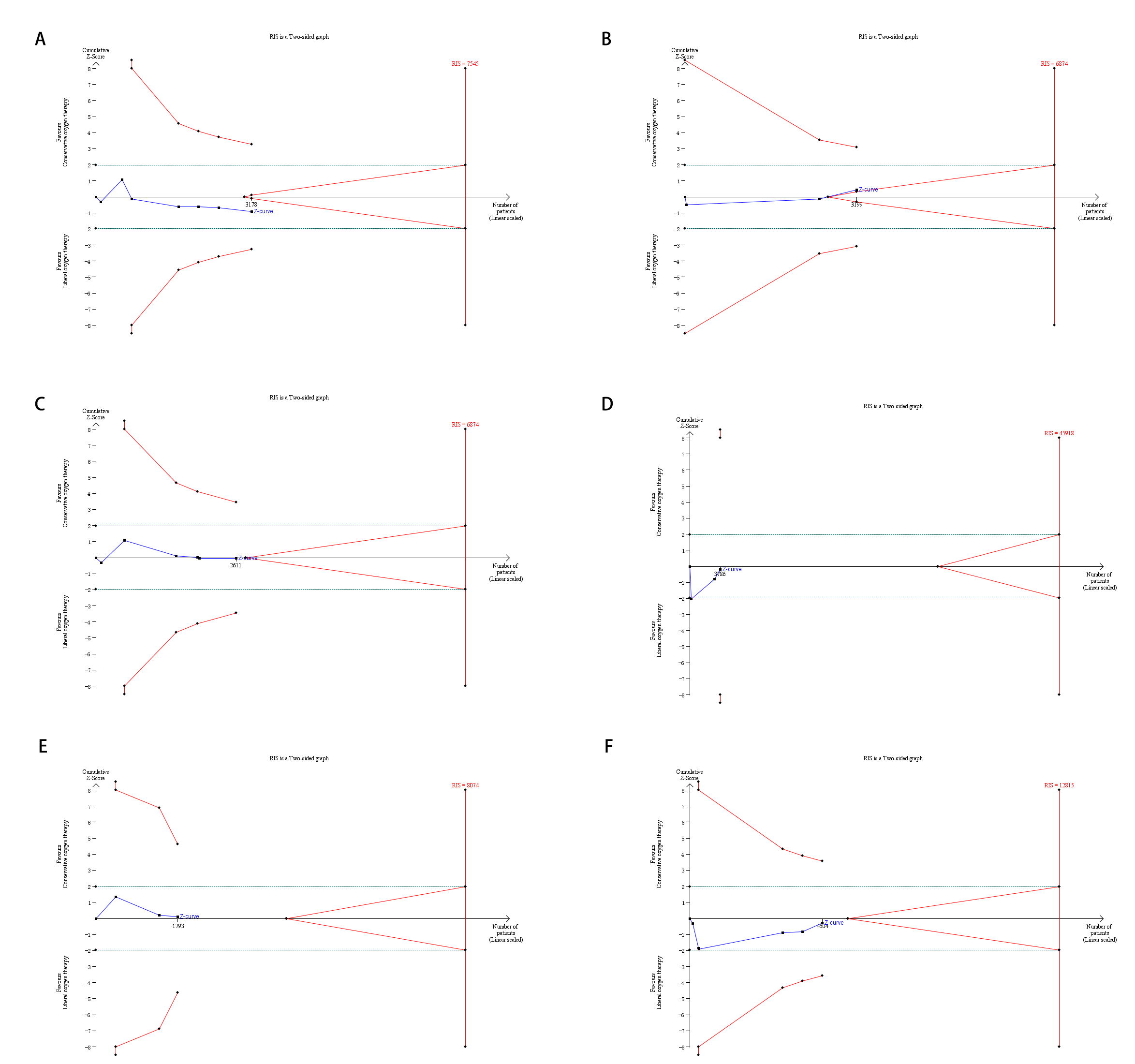
**

(A) Mixed ICU, (B) Medical ICU, (C) baseline P/F ≥ 150 mmHg, (D) baseline P/F < 150 mmHg, (E) actual PaO_2_ ≥80 mmHg in COT group, and (F) actual PaO_2_ <80 mmHg in COT group.

Figure S10. TSA of mortality at the longest follow-up (subgroup analysis) for included studies.


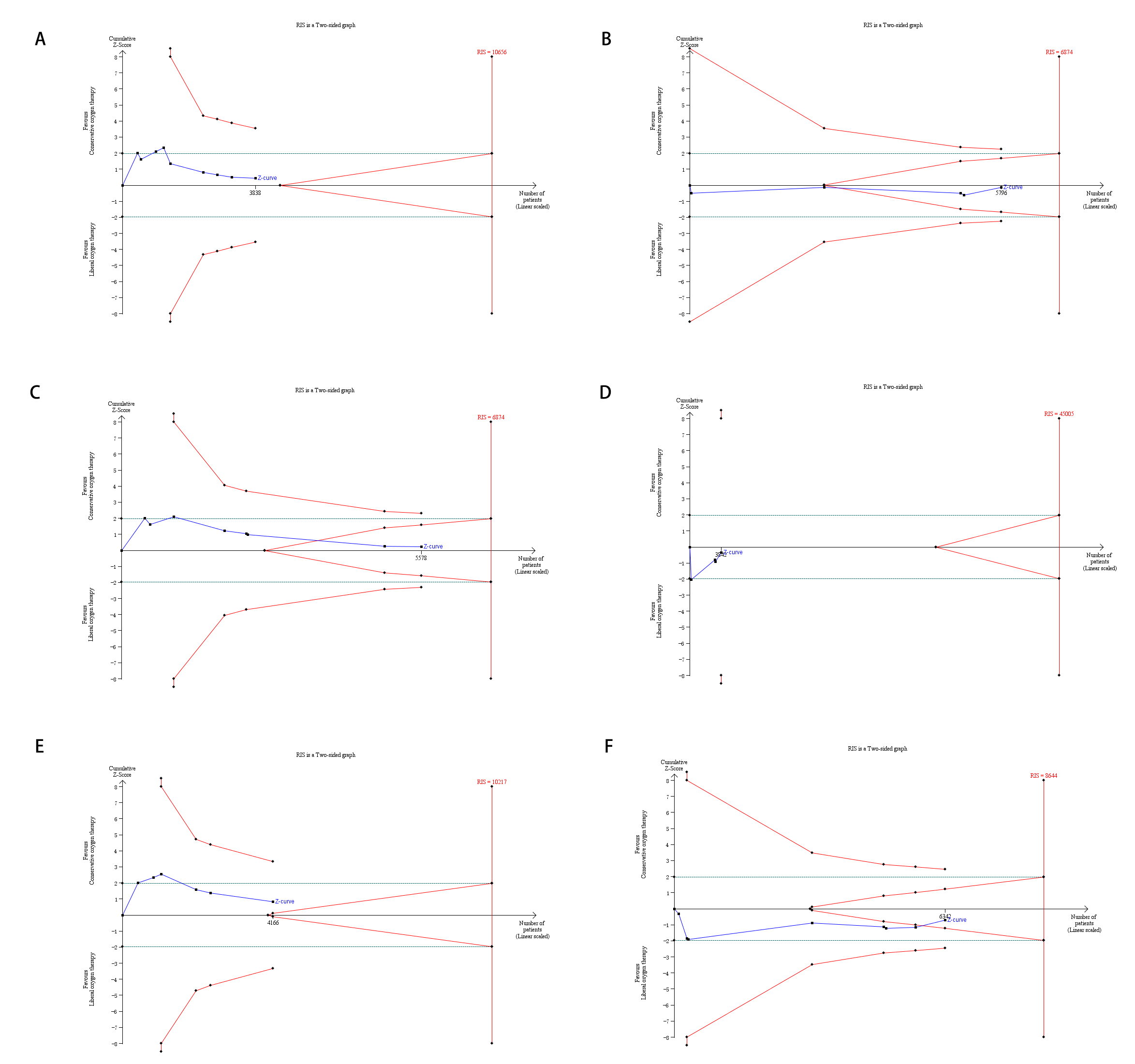


(A) Mixed ICU, (B) Medical ICU, (C) baseline P/F ≥ 150 mmHg, (D) baseline P/F < 150 mmHg, (E) actual PaO_2_ ≥80 mmHg in COT group, and (F) actual PaO_2_ <80 mmHg in COT group.

Figure S11. Secondary outcomes for included studies.

**
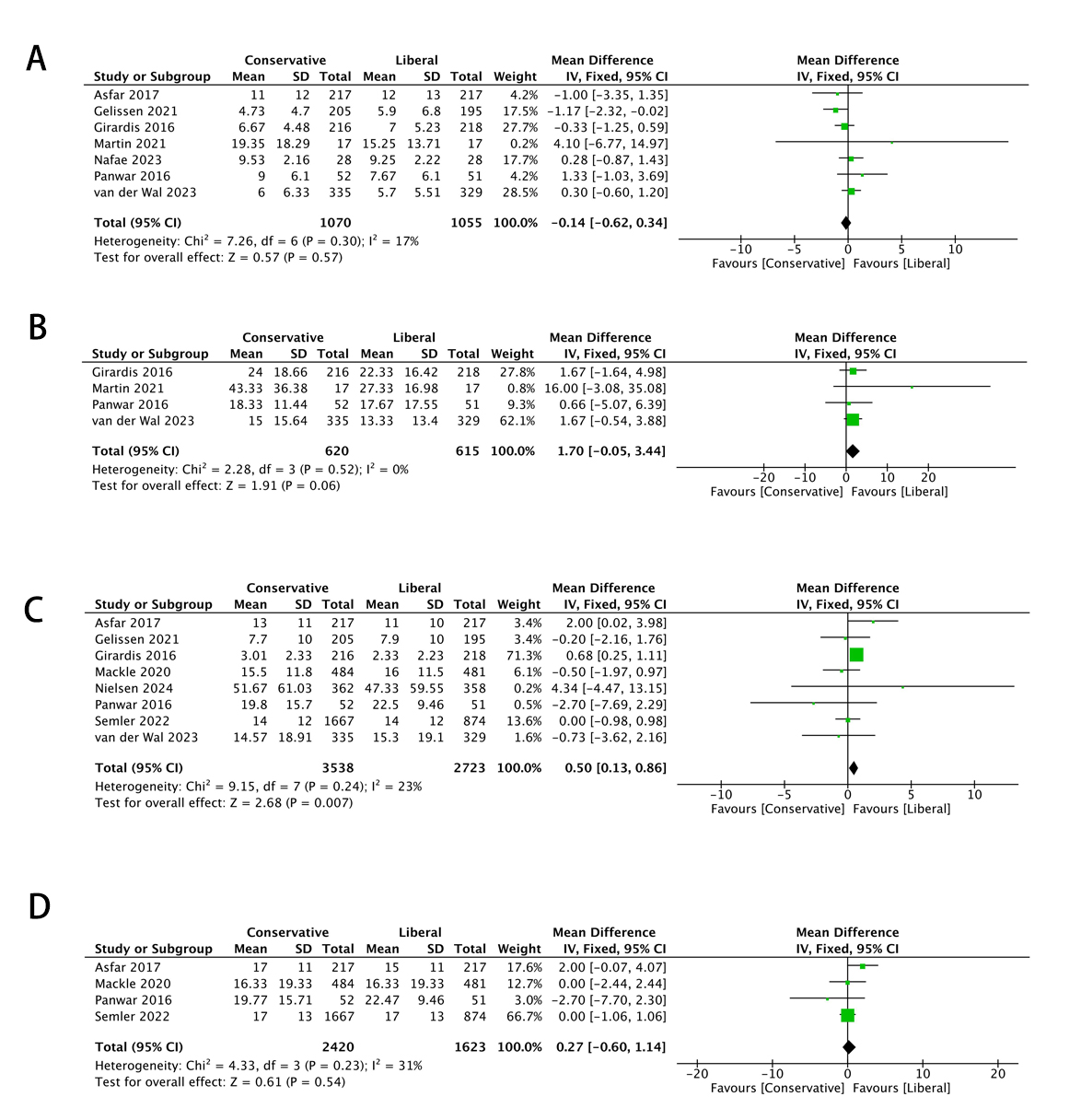
**

(A) ICU length of stay, (B) hospital length of stay, (C) mechanical ventilation-free time, and (D) vasopressor free time.

Figure S12. Funnel plot of secondary outcomes for included studies.


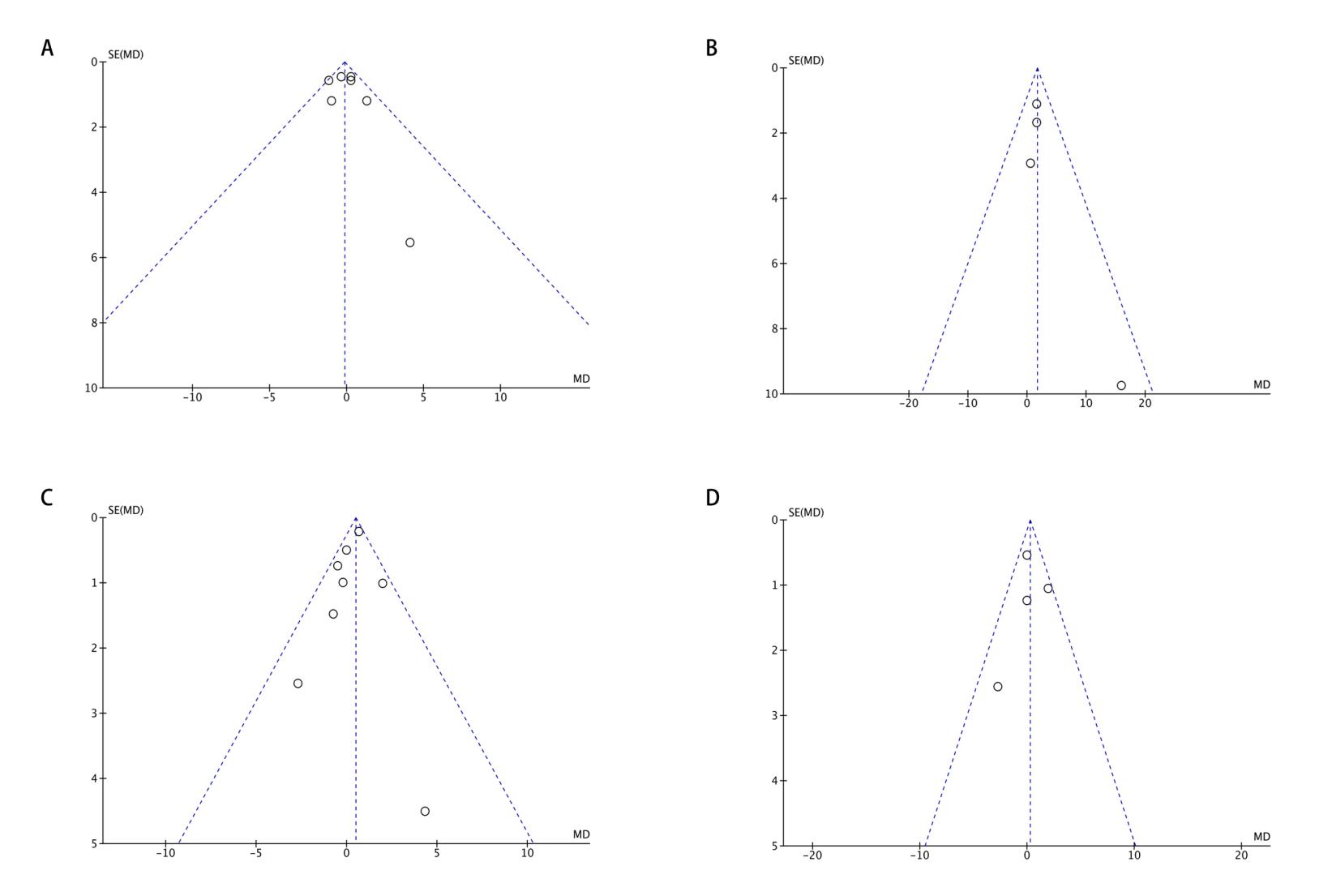


(A) ICU length of stay, (B) hospital length of stay, (C) mechanical ventilation-free time, and (D) vasopressor-free time.

Figure S13. The TSA of secondary outcomes for included studies.
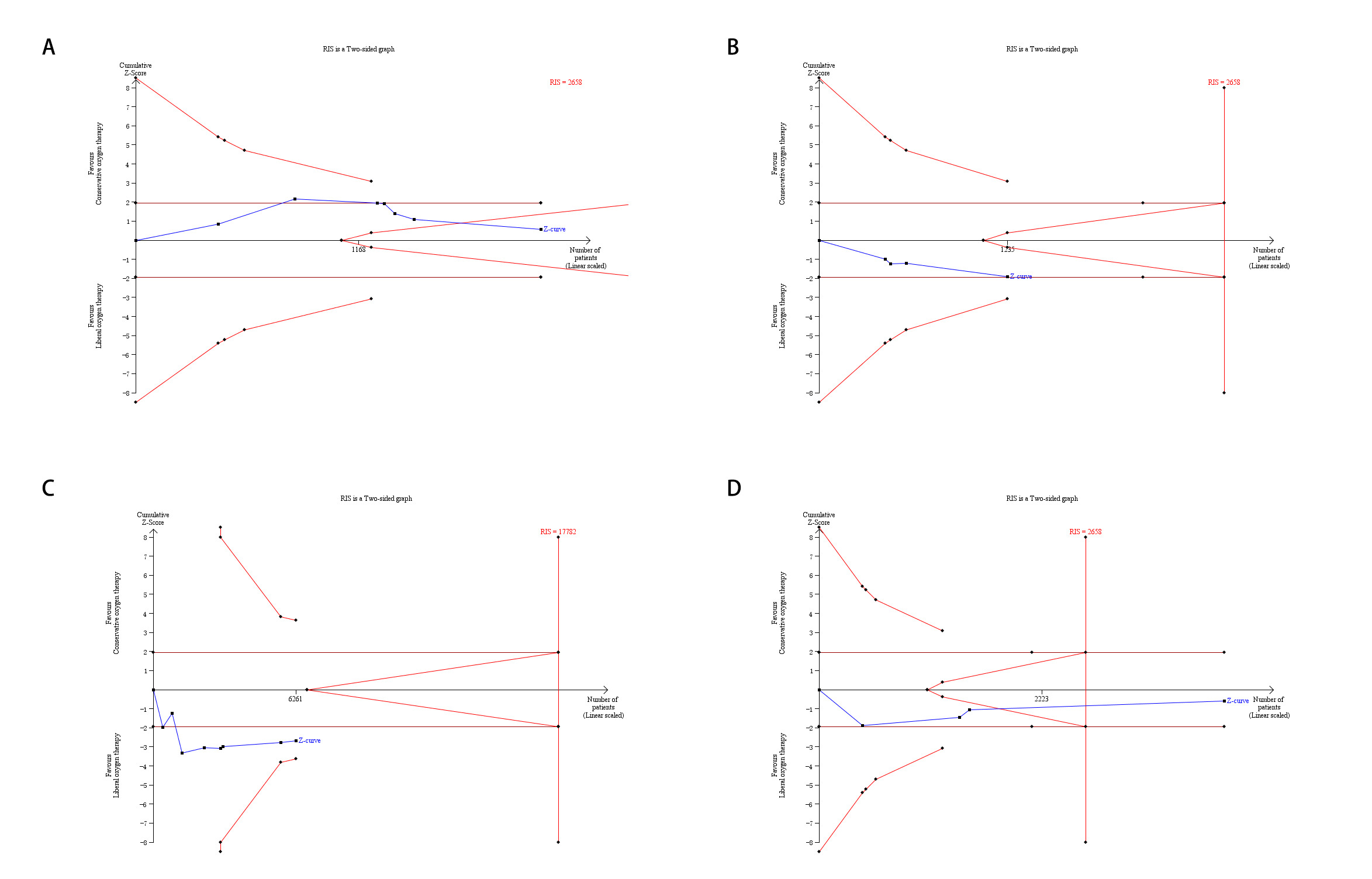


(A) ICU length of stay, (B) hospital length of stay, (C) mechanical ventilation-free time, and (D) vasopressor-free time.

Figure S14. TSA of adverse evevts for included studies.


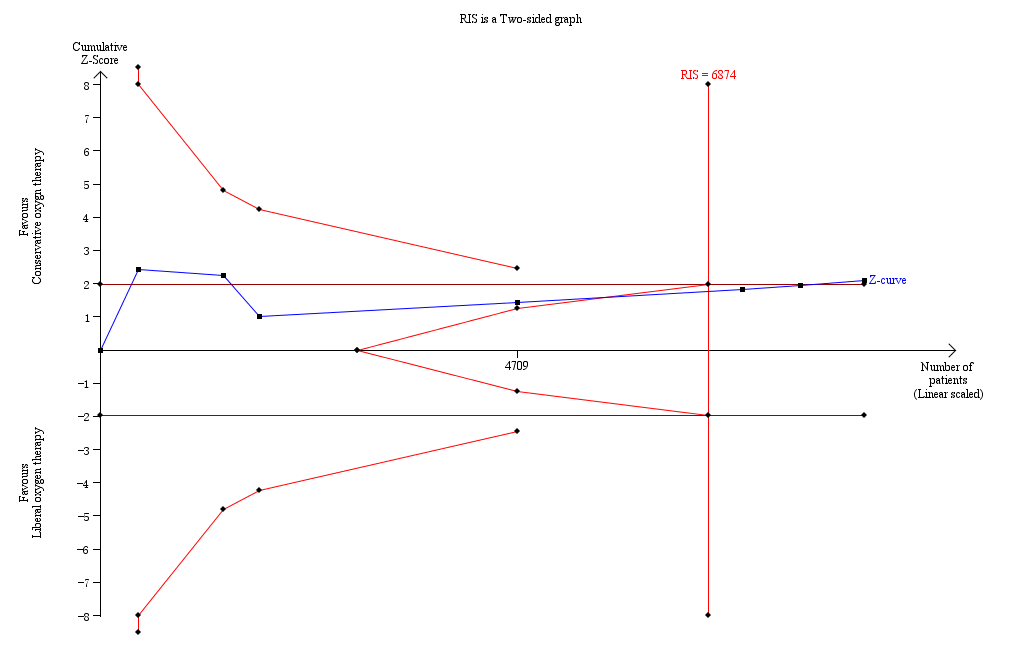


Figure S15. TSA of adverse events (subgroup analysis) for included studies.


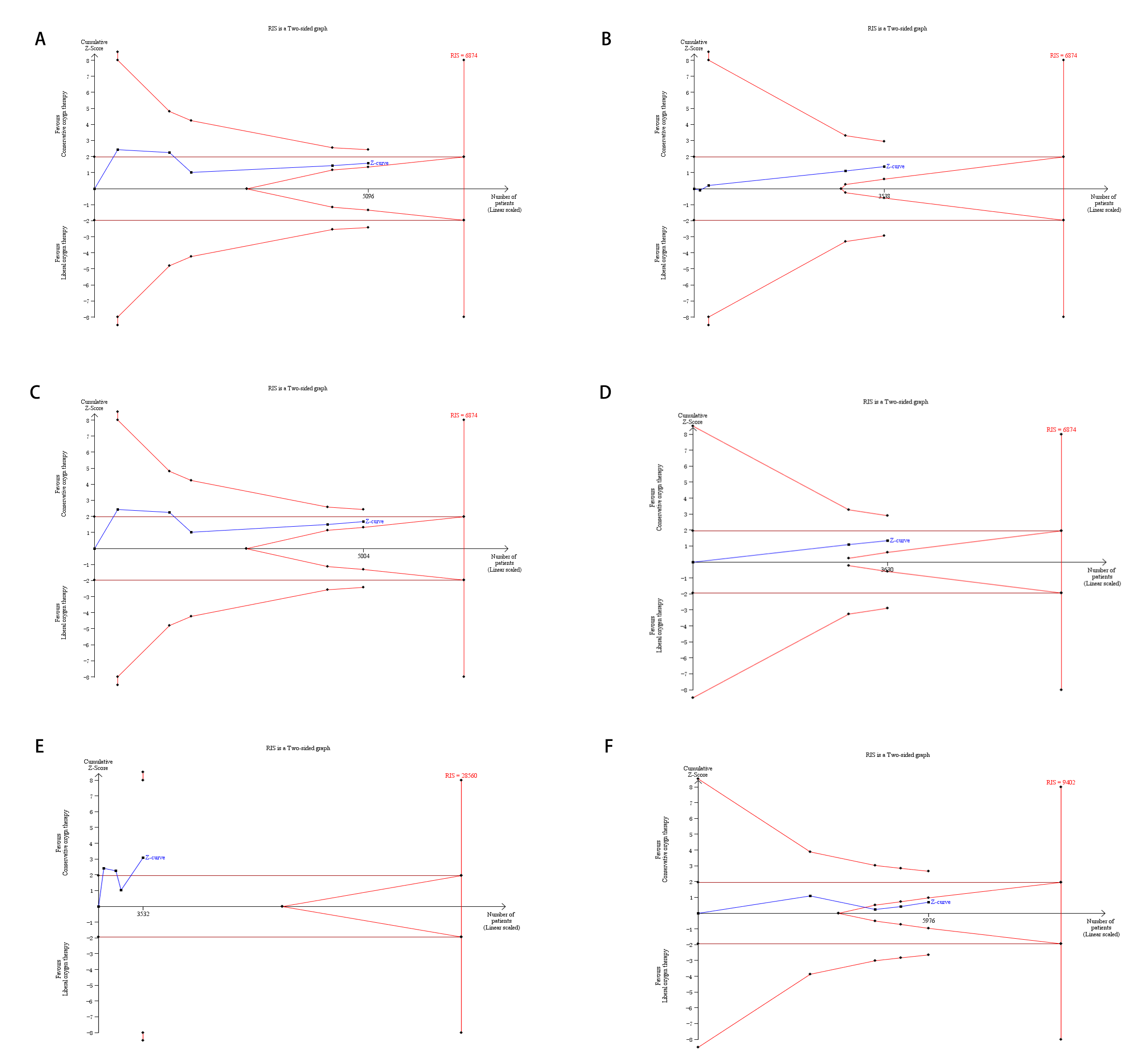


1. Mixed ICU, (B) Medical ICU, (C) baseline P/F ≥ 150 mmHg, (D) baseline P/F < 150 mmHg, (E) actual PaO_2_ ≥80 mmHg in COT group, and (F) actual PaO_2_ <80 mmHg in COT group.

Table S5 Meta-analysis of liberal versus conservative oxygen therapy in recent years

| Study | Study design included | Number of included studies（patients） | Type of patients included | Major characteristics of studies included | Conclusions |
| --- | --- | --- | --- | --- | --- |
| Chu 2018^12^ | RCT | 25 (16,037) | Mixed patients admitted to ICU included trauma, sepsis, stroke, myocardial infarction, cardiac arrest and emergency surgery | Including medical and surgical patients with the delivery method of NP, FM and IMV on admited, 43% of which with critical illness and sepsis were admitted to hospital for a surgical diagnosis. And severe hypoxamia (baseline P/F<100mmHg) were excluded with median SpO_2_ was 96.4%-96.7% at baseline. | Liberal oxygen therapy increases mortality without  improving other patient-important outcomes. |
| Hirase 2019^48^ | RCT, retrospective nested cohort study , and pilot before-and-after trial | 4 (742) | Critical illness and cardiac arrest | Study design included 2 RCTs, 1 retrospective nested cohort study, and 1 pilot before-and-after trial. And the retrospective nested cohort study only focused on patients following cardiac arrest. | Conservative oxygenation therapy resulted in significantly lower rates of ICU mortality, 28-day mortality, in-hospital mortality, and new-onset non-respiratory organ failure. |
| Barbateskovic  2019^13^ | RCT | 10 (1,458) | Mixed patients admitted to ICU included septic shock, AECOPD, OHCA, surgical diseases, stroke, traumatic brain injury | Including medical and surgical patients with the delivery method of IMVor NIV on admited. 5 trials included adults admitted to an ICU caring for patients with a range of serious health conditions and 1 to a surgical ICU. 2 trials involved adults with traumatic brain injury; 1 trial adults after cardiac arrest and resuscitation; and one trial adults with stroke. | No evidence was found for a beneficial effect of higher versus lower supplemental oxygen for adults admitted to the ICU. |
| Barbateskovic  2021^14^ | RCT | 50  (21,014) | Mixed patients admitted to ICU included stroke, septic shock, trauma, AECOPD, acute appendicitis, cardiac arrest, limb ischaemia /amputation, critical care, acute coronary syndrome, myocardial infarction, traumatic brain injury, acute abdominal surgery, acute exacerbation of asthma, acute heart failure, pneumonia, stable angina. | 35 RCTs reported the outcomes of interest to the authors. Some trials used a fixed FiO_2_, while others titrated the targeted oxygenation interval, resulting in a large span of oxygenation levels achieved in the trials. FiO_2_ in the higher oxygenation strategy ranged from 0.28 to 1.00. In the control groups, 17 trials did not apply supplemental oxygen by default. | No evidence of beneficial or harmful effects of higher versus lower oxygenation strategies in acutely ill adults was found. |
| Li 2021^15^ | RCT | 33 (17,780) | Mixed patients admitted to ICU included stroke, septic shock, trauma, ARDS, acute appendicitis, cardiac arrest, limb ischaemia, critical care, perforated viscus, myocardial infarction, sepsis, traumatic brain injury. | Patient setting varied widely：8 of the included trials were in stroke; 7 in critical care or sepsis; 3 in trauma; 7 in myocardial infarction; 4 in cardiac arrest, and 4 in emergency surgery. Some trials used a fixed FiO_2_, while others used a target interval (arterial saturation of peripheral oxygen or oxygen saturation). | No evidence was found that conservative oxygen therapy was associated with decreased mortality or incidence of disability. |
| Ni 2021^16^ | RCT, retrospective nested cohort study, pilot prospective / uncontrolled before-and-after study | 9 (5,759) | Mixed patients admitted to ICU included septic shock, ARDS, cardiac arrest, cardiac surgery, trauma, intestina ischemial, stroke, pneumonia, gastrointestinal, neurological impairment. | Study design included 6 RCTs,1 retrospective nest cohort  study and 2 prospective beforeafter studies. The enrolled patients included medical and surgical patients admitted to the ICU, the severity of whom varied greatly. | Conservative oxygen therapy did not reduce mortality but did decrease MV time, new organ failure and risk of RRT in critically ill patients |
| Zhao 2021^17^ | RCT | 8 (2,532) | Mixed patients admitted to ICU included cardiac arrest, septic shock, respiratory failure, hepatic failure, renal failure, trauma, ARDS, acute brain disease. | The oxygenation goals were classified trinary or quadruple classification system: (1) conservative (defined as PaO_2_ 55–90 mmHg, among which, PaO_2_ 55–70 mmHg was defined as far-conservative); (2) moderate (defined as PaO_2_ 90–150 mmHg); and (3) liberal (defined as PaO_2_ >150 mmHg) by constructing network geometries to visualise the comparisons between the different oxygenation goals. And the actual oxygenation levels were uesd to determine the oxygenation goal’s class. | Different oxygenation goals did not lead to different mortalities in mechanically ventilated critically ill patients. |
| Chen 2021^18^ | RCT | 7 (5,265） | ICU patients | The RCTs included subsumed under our study results, but 5 fewer papers than us. The hypoxemia severity of recruiting ICU patients varied. | Conservative oxygen therapy strategy did not improve the prognosis of the overall ICU patients. The subgroup of ICU patients with mild to moderate hypoxemia(P/F>100mmHg) had significantly lower mortality when receiving conservative oxygen therapy. |
| Dong 2022^19^ | RCT,  cohort study | 5 (1,802) | Mixed patients admitted to ICU | Study design included 4 RCTs and 1 cohort study. And the enrolled patients received mechanically ventilation. | No differences were found between the conservative and liberal groups in 28-day mortality, Mortality at 90 days, infection rates, ICU length of stay, mechanical ventilation-free days up to day 28 and vasopressor-free days up to day 28. |
| van der Wal 2023^20^ | RCT | 9 (5,807) | ICU patients | The RCTs included subsumed under our study results, but 1 fewer paper than us. All the study reports were published between 2015 and 2021 comparing a higher versus a lower oxygenation in mechanically ventilated patients focusing on the general ICU population. | No differences were found between higher and lower oxygenation strategies in Mortality at 90 days. The incidence of serious adverse events in the lower oxygenation group was low. |
| Cumpstey 2022^21^ | RCT | 8 (4,415) | Mixed patients admitted to ICU included septic shock, traumatic brain injury, ARDS, ROSC | Patient setting varied widely：2 of the included trials were in traumatic brain injury; 1 in refractory septic shock; 1 in return of spontaneous circulation after out of hospital cardiac arrest and 1 in ARDS. The severity of hypoxia varied among the enrolled patients. Some trials were receiving at least 10 litres of oxygen per minute via an open system or FIO_2_≥ 0.5 via a closed system on admission to ICU while others used a fixed FiO_2_< 0.5. | No differences were found in morbidity between high or low oxygen targets in mechanically ventilated adults. |
| Li 2023^22^ | RCT | 10 (5,429) | Mixed patients admitted to ICU included septic shock, ARDS, traumatic brain injury, OHCA, stroke, acute hypoxemic respiratory failure | 6 studies only included patients who received invasive mechanical ventilation at randomization. And 2 studies  using a fixed FiO_2_. | No beneficial or harmful effects of conservative versus liberal oxygen therapy were found on all-cause mortality at 28 days, 90 days or longest follow-up. |
| Crescioli 2022^23^ | RCT | 17 (6,592) | Mixed patients admitted to ICU included septic shock, ARDS, AECOPD, OHCA, traumatic brain injury, stroke, severe pneumonia, acute hypoxaemic respiratory failure, sugical disease. | 6 RCTs reported outcomes of interest to the authors. And the patients enrolled included medical and surgical admitted to the ICU, the severity of whom varied greatly. FiO_2_ ranged from 21% to 50% in the lower oxygenation  group, whereas in the higher oxygenation group FiO_2_ ranged from 30% to 100%. | No beneficial or harmful effects of lower versus higher oxygenation strategies were found on cognitive function, HRQoL and the standardised 6-min walking test. |
| Klitgaard 2023^24^ | RCT | 19 (10,385) | Mixed patients admitted to ICU included septic shock, AMI not accepting percutaneous coronary intervention, ARDS, AECOPD, sugical disease, OHCA, ROSC, traumatic brain injury, ischaemic and haemorrhagic stroke with no need for surgical intervention and severe pneumonia. | 17 RCTs (10,248 participants) reported the outcomes of interest to the authors. And the patients enrolled included medical and surgical patients admitted to the ICU, the severity of whom varied greatly. In 1 study the patients were assigned into lower, intermediate and higher targets according to oxygen-saturation levels. | No beneficial or harmful effects of higher versus lower oxygenation strategies were found on all-cause mortality, SAEs, quality of life, lung injuries, myocardial infarction, stroke, and sepsis at maximum follow-up. |

Abbreviations: UK: United Kingdom; RCT: Randomized controlled trial; ARDS: Acute respiratory distress syndrome; AECOPD: Acute exacerbation of chronic obstructive pulmonary disease; ROSC:Return of spontaneous circulation; OHCA:Out of hospital cardiac arrest; AMI:Acute myocardial infarction; NP: Nasal prongs; FM:Facial mask; IMV: Invasive mechanical ventilation; NIV:Non-invasive ventilation.

Table S6. PRISMA2020 checklist.

| **Section and Topic** | **Item #** | **Checklist item** | **Location where item is reported** |
| --- | --- | --- | --- |
| **TITLE** | | |  |
| Title | 1 | Identify the report as a systematic review. | Page 1 (Title) |
| **ABSTRACT** | | |  |
| Abstract | 2 | See the PRISMA 2020 for Abstracts checklist. | Page 2 (Abstract) |
| **INTRODUCTION** | | |  |
| Rationale | 3 | Describe the rationale for the review in the context of existing knowledge. | Page 3 (Introduction) |
| Objectives | 4 | Provide an explicit statement of the objective(s) or question(s) the review addresses. | Page 4 (Introduction) |
| **METHODS** | | |  |
| Eligibility criteria | 5 | Specify the inclusion and exclusion criteria for the review and how studies were grouped for the syntheses. | Page 4 (Inclusion and exclusion criteria) |
| Information sources | 6 | Specify all databases, registers, websites, organisations, reference lists and other sources searched or consulted to identify studies. Specify the date when each source was last searched or consulted. | Page 4 (Search strategy) |
| Search strategy | 7 | Present the full search strategies for all databases, registers and websites, including any filters and limits used. | Appendix page1 (Search strategy) |
| Selection process | 8 | Specify the methods used to decide whether a study met the inclusion criteria of the review, including how many reviewers screened each record and each report retrieved, whether they worked independently, and if applicable, details of automation tools used in the process. | Page 4 (Search strategy) |
| Data collection process | 9 | Specify the methods used to collect data from reports, including how many reviewers collected data from each report, whether they worked independently, any processes for obtaining or confirming data from study investigators, and if applicable, details of automation tools used in the process. | Page 5 (Data extraction and quality assessment) |
| Data items | 10a | List and define all outcomes for which data were sought. Specify whether all results that were compatible with each outcome domain in each study were sought (e.g. for all measures, time points, analyses), and if not, the methods used to decide which results to collect. | Page 4,5 (Outcomes and definition) |
|  | 10b | List and define all other variables for which data were sought (e.g. participant and intervention characteristics, funding sources). Describe any assumptions made about any missing or unclear information. | Page 4,5 (Inclusion and exclusion criteria, Data extraction and study quality) |
| Study risk of bias assessment | 11 | Specify the methods used to assess risk of bias in the included studies, including details of the tool(s) used, how many reviewers assessed each study and whether they worked independently, and if applicable, details of automation tools used in the process. | Page 5 (Data extraction and quality assessment) |
| Effect measures | 12 | Specify for each outcome the effect measure(s) (e.g. risk ratio, mean difference) used in the synthesis or presentation of results. | Page 5, 6 (Statistical analysis) |
| Synthesis methods | 13a | Describe the processes used to decide which studies were eligible for each synthesis (e.g. tabulating the study intervention characteristics and comparing against the planned groups for each synthesis (item #5)). | Page 4 (Inclusion and exclusion criteria and Figure1. |
|  | 13b | Describe any methods required to prepare the data for presentation or synthesis, such as handling of missing summary statistics, or data conversions. | Page 5, 6 (Statistical analysis) |
|  | 13c | Describe any methods used to tabulate or visually display results of individual studies and syntheses. | Page 5, 6 (Statistical analysis) |
|  | 13d | Describe any methods used to synthesize results and provide a rationale for the choice(s). If meta-analysis was performed, describe the model(s), method(s) to identify the presence and extent of statistical heterogeneity, and software package(s) used. | Page 5, 6 (Statistical analysis) |
|  | 13e | Describe any methods used to explore possible causes of heterogeneity among study results (e.g. subgroup analysis, meta-regression). | Page 4, 5, 6 (Outcomes and definition and Data extraction and quality assessment) |
|  | 13f | Describe any sensitivity analyses conducted to assess robustness of the synthesized results. | Page 4, 5, 6 (Outcomes and definition and Data extraction and quality assessment) |
| (Reporting bias assessment | 14 | Describe any methods used to assess risk of bias due to missing results in a synthesis (arising from reporting biases). | Page 5 (Data extraction and quality assessment) |
| Certainty assessment | 15 | Describe any methods used to assess certainty (or confidence) in the body of evidence for an outcome. | Page 5 (Data extraction and quality assessment) |
| **RESULTS** | | |  |
| Study selection | 16a | Describe the results of the search and selection process, from the number of records identified in the search to the number of studies included in the review, ideally using a flow diagram. | Page 6 (Study selection, and Figure 1) |
|  | 16b | Cite studies that might appear to meet the inclusion criteria, but which were excluded, and explain why they were excluded. | Page 6 (Study selection, Figure 1) |
| Study characteristics | 17 | Cite each included study and present its characteristics. | Page 6, 7 (Study description and quality assessment and Table1); Appendix page 3-11 (Tables S1–4) |
| Risk of bias in studies | 18 | Present assessments of risk of bias for each included study. | Page 6, 7 (Study description and quality assessment); Appendix page 12-14, 21 (Figure S1-3, S10) |
| Results of individual studies | 19 | For all outcomes, present, for each study: (a) summary statistics for each group (where appropriate) and (b) an effect estimate and its precision (e.g. confidence/credible interval), ideally using structured tables or plots. | Page 7, 8 (Outcomes and Figure 2-4); Appendix page 15-22 (Figure S4-11) |
| Results of syntheses | 20a | For each synthesis, briefly summarise the characteristics and risk of bias among contributing studies. | Page 6, 7 (Study description and quality assessment); Appendix page 12-14, 23 (Figure S1-3, S12) |
|  | 20b | Present results of all statistical syntheses conducted. If meta-analysis was done, present for each the summary estimate and its precision (e.g. confidence/credible interval) and measures of statistical heterogeneity. If comparing groups, describe the direction of the effect. | Page 7, 8 (Outcomes and Figure 2-4); Appendix page 15-22 (Figure S4-11) |
|  | 20c | Present results of all investigations of possible causes of heterogeneity among study results. | NA |
|  | 20d | Present results of all sensitivity analyses conducted to assess the robustness of the synthesized results. | Page 7, 8 (Outcomes and Figure 2-4); Appendix page 15-22 (Figure S4-11) |
| Reporting biases | 21 | Present assessments of risk of bias due to missing results (arising from reporting biases) for each synthesis assessed. | Page 6, 7 (Study description and quality assessment) |
| Certainty of evidence | 22 | Present assessments of certainty (or confidence) in the body of evidence for each outcome assessed. | Page 7, 8 (Outcomes) |
| **DISCUSSION** | | |  |
| Discussion | 23a | Provide a general interpretation of the results in the context of other evidence. | Page 8-11 (Discussion) |
|  | 23b | Discuss any limitations of the evidence included in the review. | Page 11 (Discussion) |
|  | 23c | Discuss any limitations of the review processes used. | Page 11 (Discussion) |
|  | 23d | Discuss implications of the results for practice, policy, and future research. | Page 10-11 (Discussion) |
| **OTHER INFORMATION** | | |  |
| Registration and protocol | 24a | Provide registration information for the review, including register name and registration number, or state that the review was not registered. | Page 3-4 (Materials and methods) |
|  | 24b | Indicate where the review protocol can be accessed, or state that a protocol was not prepared. | Page 3-4 (Materials and methods) |
|  | 24c | Describe and explain any amendments to information provided at registration or in the protocol. | NA |
| Support | 25 | Describe sources of financial or non-financial support for the review, and the role of the funders or sponsors in the review. | Page 12 (Funding) |
| Competing interests | 26 | Declare any competing interests of review authors. | Page 12 (Competing interests) |
| Availability of data, code and other materials | 27 | Report which of the following are publicly available and where they can be found: template data collection forms; data extracted from included studies; data used for all analyses; analytic code; any other materials used in the review. | Page 12 (Availability of data and materials) |

*From:*  Page MJ, McKenzie JE, Bossuyt PM, Boutron I, Hoffmann TC, Mulrow CD, et al. The PRISMA 2020 statement: an updated guideline for reporting systematic reviews. BMJ 2021;372:n71. doi: 10.1136/bmj.n71

For more information, visit: <http://www.prisma-statement.org/>
